# Peer violence perpetration and victimization: Prevalence, associated factors and pathways among 1752 sixth grade boys and girls in schools in Pakistan

**DOI:** 10.1371/journal.pone.0180833

**Published:** 2017-08-17

**Authors:** Rozina Karmaliani, Judith Mcfarlane, Rozina Somani, Hussain Maqbool Ahmed Khuwaja, Shireen Shehzad Bhamani, Tazeen Saeed Ali, Saleema Gulzar, Yasmeen Somani, Esnat D. Chirwa, Rachel Jewkes

**Affiliations:** 1 School of Nursing and Midwifery, Aga Khan University, Karachi, Pakistan; 2 Texas Woman’s University, Denton, Texas, United States of America; 3 South African Medical Research Council, Pretoria, South Africa; University of Westminster, UNITED KINGDOM

## Abstract

**Background:**

Child peer violence is a global problem and seriously impacts health and education. There are few research studies available in Pakistan, or South Asia. We describe the prevalence of peer violence, associations, and pathways between socio-economic status, school performance, gender attitudes and violence at home.

**Methods:**

1752 children were recruited into a cluster randomized controlled trial conducted on 40 fairly homogeneous public schools (20 for girls and 20 for boys), in Hyderabad, Pakistan. This was ranging from 20–65 children per school. All children were interviewed with questionnaires at baseline.

**Results:**

Few children had no experience of peer violence in the previous 4 weeks (21.7% of girls vs.7% of boys). Some were victims (28.6%, of girls vs. 17.9% of boys), some only perpetrated (3.3% of girls vs. 2.5%) but mostly they perpetrated and were victims (46.4%.of girls vs 72.6%. of boys). The girls’ multivariable models showed that missing the last school day due to work, witnessing her father fight a man in the last month and having more patriarchal gender attitudes were associated with both experiencing violence and perpetration, while, hunger was associated with perpetration only. For boys, missing two or more days of school in the last month, poorer school performance and more patriarchal attitudes were associated with both victimization and perpetration. Witnessing father fight, was associated with peer violence perpetration for boys. These findings are additionally confirmed with structural models.

**Discussion:**

Peer violence in Pakistan is rooted in poverty and socialization of children, especially at home. A critical question is whether a school-based intervention can empower children to reduce their violence engagement in the context of poverty and social norms supportive of violence. In the political context of Pakistan, reducing all violence is essential and understanding the potential of schools as a platform for intervention is key.

## Introduction

Peer violence has been defined as a repeated act that is intentionally exercised to harm others on purpose, it is violence that is patterned rather than an isolated occurrence [1]. It has further been defined as ‘unwanted, aggressive behavior among school-aged children that involves a real or perceived imbalance of power’ [2]. Peer violence may be direct or indirect. Direct violence includes physical aggression, threats, and name-calling. Whereas, indirect violence includes spreading of rumors, backstabbing, and exclusion [3, 4]. Peer violence can thus be of physical, verbal and social, and may be sexual [5]. Although it has been described in a great range of countries it has not been the subject of much previous research in Pakistan.

The estimated prevalence of peer violence among children and adolescent in schools varies between countries from less than 10% to over 65% of children exposed globally [6]. A recent review of South East Asian countries (Indonesia, Malaysia, Singapore, Philippines), reported the range of prevalence of peer violence at school is from 7% to 59%, with Philippines being the highest [7]. One study from India reported that 60% of children had been bullied [8]. In the United States it is estimated that 90% of students of grade 4-8^th^ are victim of peer violence and as many as 160,000 school children are estimated to avoid going to school regularly because of the fear of being harassed by other students [9]. Adults often fail to protect children from peer violence, and evidence shows that when school teachers and the school administration is not willing to support the victims, this may lead to more intense peer violence [10].

Peer violence among children has serious consequences for children’s health and academic performance. It results in fatigue, insomnia, and poor concentration at school. It negatively impacts on a victim’s self-esteem, which leads to depression, psychosomatic problems, loss of confidence, distrust of others, social anxiety, suicide and even homicide [11, 12]. Those who perpetrated have been described as being at heightened risk of violence and substance abuse and truancy [12]. A study of psychological symptoms among school children ages 11, 13 and 15 from various schools of Europe and North America indicates that temperament issues, nervousness, sleep disturbance, loneliness, and hopelessness are the significant psychological symptoms faced by students due to bullying within the schools [13]. Another study revealed that, around 40% of the children reported being sad and depressed about being bullied [8].

A number of factors have been described as associated with peer violence. A recent systematic review found 85 articles on predictors of peer violence perpetration, but none came from central, south or south-east Asia. The associated factors and drivers of violence described included individual level factors, family level drivers and school-related drivers [14]. Some of the research has focused on drivers located in the family environment where there is transmission of anti-social behavior across generations, through social learning, transmission of attitudes around acceptability of the use of violence and re-enactment [15]. Connections between poverty and bullying have provided mixed findings and parental involvement in children’s schooling and life is protective. Bullying is commonly associated with having less interest in schooling and poorer performance, having more friends and more positively endorsing violence behavior. School environments which affirm students’ rights and are more democratic are protective [16].

Although, much of the research on violence among young teenagers does not link it to gender and to subsequent gender violence experience or perpetration, there are exceptions [17]. Peer violence is gendered, to the extent that it is more highly prevalent among boys than girls and often rooted in ideas of socially appropriate boyish behavior [18]. Moreover, engagement of men and boys in some forms of peer violence, especially in gangs or other delinquent peer associations, has been shown to be strongly associated to subsequent perpetration of sexual violence against women [19, 20]. The growing evidence on the interconnectedness of different forms of violence leads us to believe that the types of social skills and empowerment interventions which would be important in reducing peer violence would also be beneficial in reducing other forms of gender-based violence in later life.

This was the rationale for including an evaluation of the NGO Right To Play’s Red Ball Child Play intervention in Pakistan in the portfolio of interventions to be evaluated under the UK-Aid funded What Works To Prevent Violence Against Women and Girls? Global Programme. As far as we know, this is the first randomized controlled trial of an intervention that seeks to reduce peer violence from a low or middle income country setting [21]. In this paper we draw on data from the baseline of this cluster randomized controlled trial. The aims of the paper are to describe the prevalence of peer violence among sixth graders in schools of Pakistan and associations and pathways between socio-economic status, school performance, mental health, gender attitudes and violence at home.

## Methods

The data were from the baseline of the cluster randomized controlled trial conducted to evaluate the intervention Red Ball Child Play of the NGO Right to Play. The trial was conducted on 40 fairly homogeneous schools (20 for girls and 20 for boys), in Hyderabad, Sindh province of Pakistan. We selected age 11 to 12, the 6^th^ grade, for the initial phase of the research. Inclusion criteria for schools were that they should be single gender public middle schools with an outside playground or indoor space in which games can be played, and to have 25 or more students in the grade 6 class and giving consent to participate. To reduce contamination between arms, we only included schools that were more than 1 km away from the nearest other included schools of that gender. For children, in addition to being in 6^th^ grade, they had to provide parental consent and themselves agree to the research. They also had to read the national language of Pakistan Urdu or the provincial language Sindhi. In total 1752 children were recruited into the study. In small schools we invited the whole 6^th^ grade to participate but if the school was large the grade was often divided into 2–3 sections and then we invited just one. The number of children per school ranged from 20–65. Details of the methods of sampling and recruitment are detailed elsewhere [22].

All children were interviewed with standard questionnaires. The instruments were partially self-administered. Although the instrument was written to a 6th grade reading level, many children had difficulty reading and so we formed clusters on one researcher and four children and the researcher read each question and the children marked the response. The questionnaires were double entered into a SPSS data file and discrepancies resolved.

Children were asked their age, how many siblings they had and socio-economic status was assessed through two questions which asked about the frequency of going to be without dinner and school without breakfast. These questioned were summed to give a food insecurity/hunger score. School performance was assessed through four questions which asked about performance in maths, science, language and Pakistan studies (below average, average and above average). These were summed to give an overall school performance score. The number of days absent from school was asked, whether a grade had been repeated, as well as a series of questions inquiring about the main reason for the last absence (working at home, working outside the home, bullying, not having done homework). Two questions asked about abuse of the child’s mother in the previous month: one about physical violence from her husband and the other about physical violence from any family member. A variable was derived as a measure of any abuse of the mother by an in-law or her husband. A question was also asked if the child had seen or heard his father fighting physically with another man in the last month. A gender attitudes scale was used with eight items. The gender attitudes questions had a 4 point Likert response scale and were developed for the setting. They required a response to the following statements: I think girls in our family should go to school; I think the husbands in your family should give permission to give their wives to go to the clinic; I think the husbands in the family should listen to their wives’ opinion on schooling; I think the wives in the family should have a say in how money in their family is spent; I think the wives in the family should be able to ask a religious scholar about issues; I think the husbands in the family should respect the opinion of their wives on matters related to income generating work; I think a husband in the family should be kind and caring toward the women in his family; and I think that the wives in our family should always obey their husbands. The questions were summed to give a gender (inequitable) attitudes score.

The Peer Victimization Scale is a 16-item measure with 4 subscales, each with 4 questions, assessing physical and verbal victimization, social manipulation, and property attacks in the previous 4 weeks [23]. Designed for youth, age 11–17, respondents were asked over the last 4 weeks, how often (i.e., never, once, a few times (2–3) or many times (4 or more) an event happened to them (i.e. victimization). Scale scores were computed by summing item responses. Scores on the total scale have a possible range of 0 to 48. Cronbach’s alpha was 0.86 for both girls and boys.

A Peer Perpetration Scale was developed for the study based on the peer victimization scale. We asked the same 16-items of the peer victimization scale with the wording adjusted to measure perpetration[23]. It has the same 4 subscales and scoring. Cronbach’s alpha was 0.86 for both girls and 0.89 for boys.

This study was approved by Ethical Review Committee (ERC) of Aga Khan University (AKU) and the Ethics Committee of the Medical Research Council of South Africa. After receiving permission from the Department of Education, schools gave written consent to participate in the study. The study was explained to children and they were sent home with an information sheet and consent form for their parents to complete and sign if they agreed to participation in the research. After receiving signed consent forms from parents; students were invited to give consent themselves.

### Data analysis

The data was analyzed using Stata 13.0 and all analyzed took into account the structure of the sample, with the students clustered into schools. Variables were summarized as percentages (or means), and the statistical test of difference between categories was a Pearson chi square test or t-test. The main outcome used in the analyses with a three level peer violence variable. First we scored the 16 victimization and 16 perpetration questions by adding the items. Then we used the CDC definition [24] of peer violence which groups zero and one type/episode of violence together as ‘no/low’ violence and considered an act of victimization or perpetration to have ‘occurred’ if there had been more than one episode/type. We then derived a three level variable with levels that we refer to as ‘no violence’ (a group CDC would call ‘no/low’, n = 55 boys and 194 girls), ‘victimization only’ (n = 146 boys and n = 265 girls) and ‘perpetration (with or without victimization)’ (n = 621 boys and n = 471 girls). In order to be in the ‘victimization only’ category there should have been no more than one type/episode of perpetration, but the perpetration category included many children who reported both victimization and perpetration.

In order to determine factors associated with victimization and perpetration among girls and boys, multinomial regression models were built with the three level peer violence variable as the outcome and candidate independent variables were the social and demographic characteristics of the children, school attendance and performance variables, violence at home and gender attitudes. The variables were entered into the model and sequential backwards elimination was used, removing those with the largest value for p first until the final variables were retained at p< or = 0.05.

Structural Equation Modeling (SEM) was conducted on the data using Stata 13.0, separately for girls and boys. The models’ outcome was a latent variable for peer perpetration based on the sixteen peer perpetration variables and for each model those who had just experienced victimisation were excluded from the analysis, giving a total sample size of 665 for the girls perpetration model and 676 for the boys one. For model building we prepared each latent variable and used confirmatory factor analysis to optimize the indicators of each latent variable. The standardized coefficients were examined. Where factor loadings were weak (<0.3), variables were dropped to optimize measurement. The goodness of fit was tested for each latent variable, suggested modification indices were examined and those that fitted theoretically were included such that the fit was optimized through errors being allowed to co-vary. The correlation between each hypothesized latent variable and the peer perpetration latent variable were then tested by building variable pairs. All associations were tested by running a full-information maximum likelihood method with adjustment for missing values.

**Fig 1:**
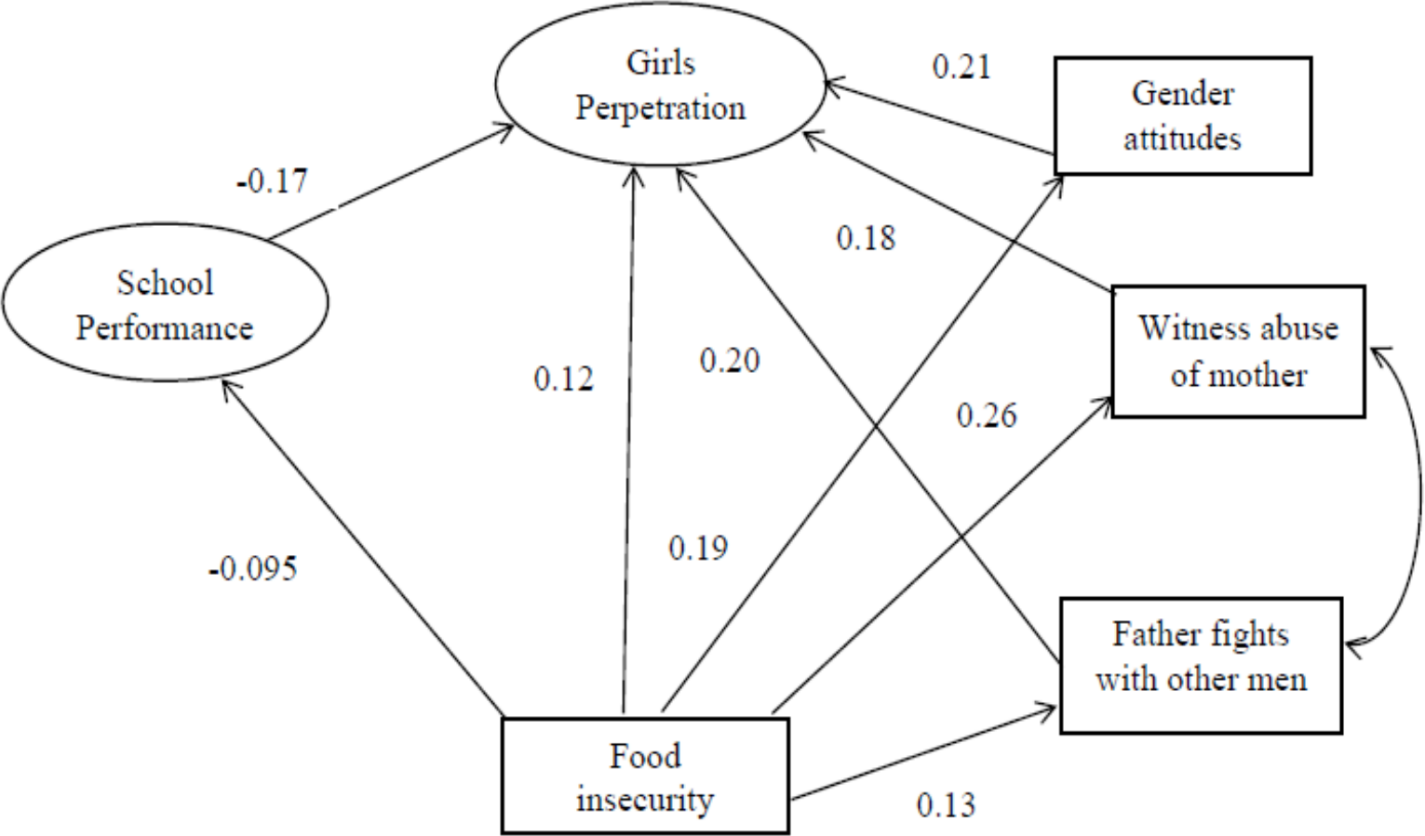
Final structural models of factors associated with peer violence perpetration for girls.

**Fig 2:**
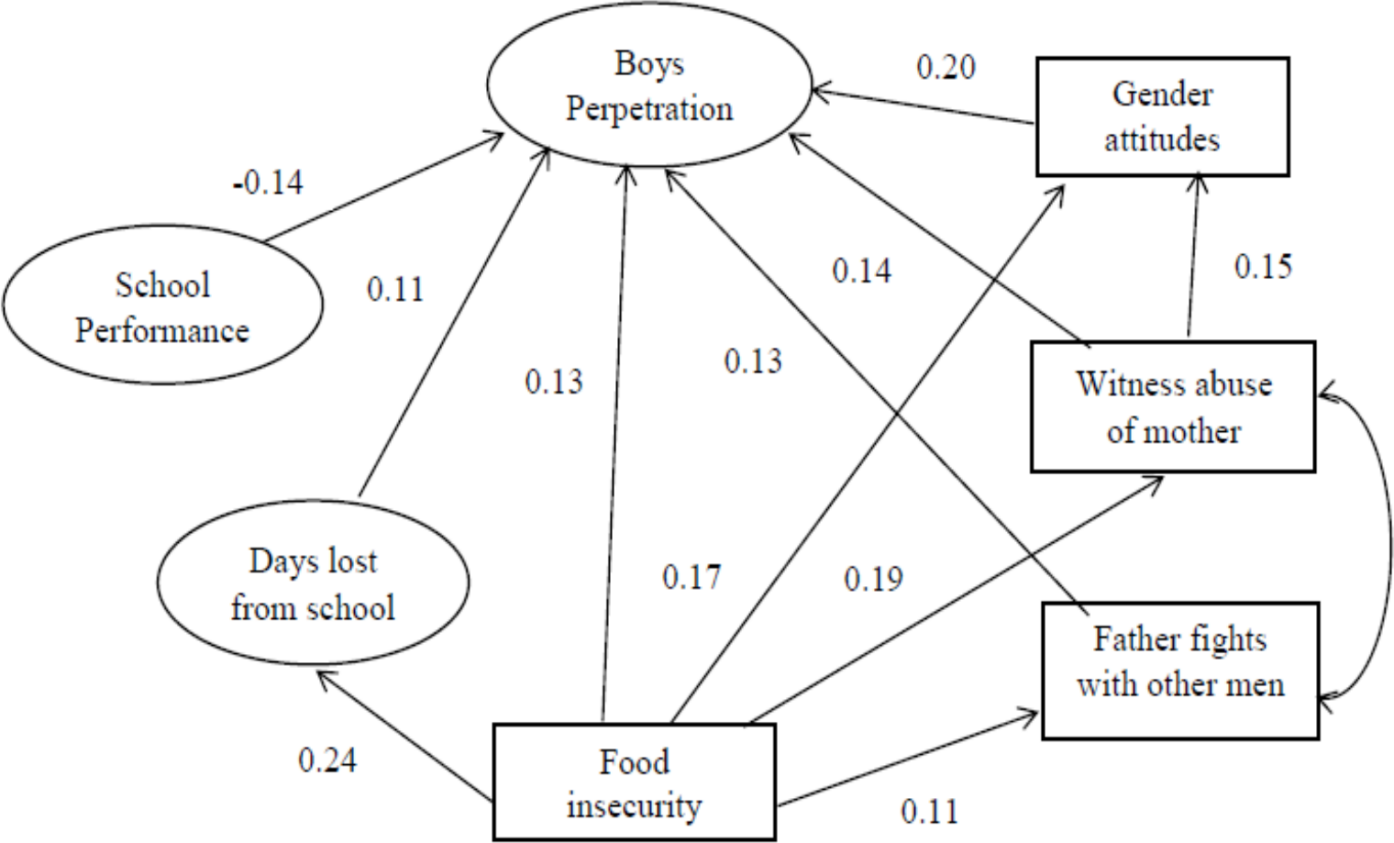
Final structural models of factors associated with peer violence perpetration for boys.

As a next stage, a measurement model was fitted with the latent variables allowed to freely correlate. To assess model fit of the observed data, we used the comparative fit index (CFI) (>0.95); Tucker-Lewis Index (TLI) (>0.9) for acceptable fit and (>0.95) as indicative of good fit [25]; and root mean square error of approximation (RMSEA) (of 0.05 or less) [26, 27]. The model chi-square test was examined, but it was not used in assessing model fit because it has unsatisfactory properties, such as inflation with large sample sizes [27]. We systematically deleted non-significant paths. Next the structural relations among our latent variables were examined. The final structural models are presented in Figs 1 & 2, with the significance of individual paths and final factor loadings indicated. For boys: CFI = .984; TLI = .982; RMSEA = .015. For girls: CFI = .976; TLI = .968; RMSEA = .024.

## Results

The baseline survey was completed for 1752 children from 40 schools (20 girls and 20 boys), of which 930 were girls and 822 were boys. 90.8% of boys and 75.3% of girls had experienced more than one instance of violence victimization and 75.0% of boys and 49.9% of girls disclosed perpetration of more than one instance of violence in the prior four weeks. There was considerable overlap between experience of victimization and perpetration. In girls, 46.4% reported both, 3.3% only perpetration, 28.6% only experience of victimization and 21.7% neither. In boys 72.6% had experienced/done both, 2.5% had only perpetrated and 17.9% had only been victims. Only 7% were in neither category.

Table 1 shows the characteristics of girls and boys by disclosure of perpetration and victimization. The mean age of the both genders (12 years) for perpetration and victimization was similar. Although boys who perpetrated peer violence were younger than those who had not engaged in violence (p = 0.05) and the mean age of girls who were victimized was younger than that of other girls (<0.001). Girls who were not victimized nor had perpetrated peer violence had significantly fewer brothers than those who had, but there was no other difference between peer violence group, gender and number of siblings. There were significant differences in food security, a measure of socio-economic status, by peer victimization and perpetration. Most notably, boys and girls who perpetrated peer violence scored higher on food insecurity than those who did not. Overall 26% of boys and 18% of girls reported having gone to bed without dinner due to lack of food in the previous month and 24% of boys and 18% of girls had gone to school without breakfast. Girls who were victimized and perpetrated and boys who perpetrated scored higher on the hunger measure than those who did not.

**Table 1:** Social and demographic characteristics of girls and boys by engagement in and experience of peer violence.

	BOYS							GIRLS						
	Neither		Victimisation only		Perpetration (+/- victimisation)			Neither		Victimisation only		Perpetration (+/- victimisation)		
Social Demographics	n	mean (SD)	n	mean (SD)	n	mean (SD)	P value	n	mean (SD)	n	mean (SD)	n	mean (SD)	P value
Age	55	12.7(1.5)	145	12.6(1.6)	621	12.5(1.5)	0.053	194	12.3(1.4)	265	12.4(1.3)	468	12.2(1.4)	<0.001
# of brothers	55	2.9(1.9)	146	2.7(1.7)	618	2.7(1.6)	0.461	194	2.1(1.3)	265	2.2(1.3)	471	2.3(1.4)	<0.001
# of sisters	55	2.3(1.7)	146	2.0(1.6)	615	2.2(1.5)	0.086	194	2.6(1.8)	265	2.6(1.8)	471	2.7(1.8)	0.526
Hunger score	55	0.4(1.0)	146	0.4(0.8)	621	0.7(1.1)	<0.001	194	0.3(0.7)	265	0.4(0.9)	471	0.6(1.1)	<0.001
**Go to school without breakfast**		%		%		%			%		%		%	
Never	46	83.6	123	84.2	456	73.4	0.039	175	90.2	222	83.8	365	77.5	0.003
sometimes	8	14.5	19	13	123	19.8		12	6.2	29	10.9	77	16.3	
Often	1	1.8	4	2.7	42	6.8		7	3.6	14	5.3	29	6.2	
**Go to bed without dinner**														
Never	46	83.6	124	84.9	439	70.7	0.012	180	92.8	228	86	354	75.2	0.013
sometimes	7	12.7	19	13	137	22.1		10	5.2	29	10.9	93	19.7	
Often	2	3.6	3	2.1	45	7.2		4	2.1	8	3	24	5.1	

Overall 25.5% of boys and 21.2% of girls had repeated a school grade. 86% of boys and 82.4% of girls had missed 2 or more days of school in the previous month. The distribution of factors related to school attendance and performance by engagement in peer violence, are shown in Table 2. There was no difference in self-reported school performance, grade having been repeated and absence among girls by whether they had been a victim or perpetrator of violence, but among boys school performance was poorer and school absence greater among those who were victims and perpetrated. Among girls, the proportion who took the “last day off due to working” was much higher amongst those experiencing violence as victims and perpetrating. Among boys, there was no difference in the reason for the last day being missing from school by peer violence category.

**Table 2:** School attendance and performance, gender attitudes and violence experience at home of girls and boys by engagement in and experience of peer violence.

		BOYS							GIRLS						
		Neither		Victimisation only		Perpetration (+/- victimisation)			Neither		Victimisation only		Perpetration (+/- victimisation)		
		n	%	n	%	n	%	P value	n	%	n	%	n	%	P value
Ever repeated a grade	Yes	9	16.7	29	20	170	27.5	0.045	36	18.7	58	22.1	102	21.7	0.621
	No	45	83.3	116	80	448	72.5		157	81.3	205	77.9	368	78.3	
Absence from school in last 4 weeks	2 or more	33	60	125	86.8	541	88.1	<0.001	154	79.8	218	82.3	392	83.6	0.49
	none	22	40	19	13.2	73	11.9		39	20.2	47	17.7	77	16.4	
Last day off due to working	Yes	7	13.7	33	24.3	155	27.2	0.266	16	8.9	45	18.1	69	16.5	0.01
	no	44	86.3	103	75.7	415	72.8		164	91.1	203	81.9	349	83.5	
Last day off due to working outside the home	Yes	3	5.9	7	5.1	52	9.1	0.186	2	1.1	5	2	8	1.9	0.484
	no	48	94.1	129	94.9	519	90.9		178	98.9	242	98	410	98.1	
Last day off due to being afraid to attend because of bullying	Yes	3	5.9	8	5.9	45	7.9	0.524	1	0.6	8	3.2	15	3.6	0.082
	no	48	94.1	128	94.1	523	92.1		179	99.4	240	96.8	403	96.4	
Last day off due to not having done homework	Yes	12	24.5	42	30.9	163	29	0.684	43	24	51	20.6	106	25.3	0.459
	no	37	75.5	94	69.1	399	71		136	76	197	79.4	313	74.7	
School performance (mean score (SD))		55	10(1.7)	146	9.6(1.8)	621	9.1(1.8)	0.171	194	9.8(1.6)	265	9.7(1.7)	471	9.4(1.8)	0.06
Any abuse of mother	Yes	0	0	8	5.5	98	15.8	0.046	1	0.5	13	4.9	63	13.4	<0.001
	no	55	100	138	94.5	522	84.2		193	99.5	252	95.1	407	86.6	
Seen or heard father to have physical fight with another man	Yes	2	3.6	21	14.5	188	30.3	<0.001	12	6.2	32	12.1	122	26	0.002
	no	53	96.4	124	85.5	432	69.7		182	93.8	233	87.9	347	74	
Gender inequitable attitude scale (8 items mean (SD))		55	9.8(3.3)	146	11(3.7)	621	12(4.3)	<0.001	194	9.8(3.1)	265	10(3.5)	471	11(3.8)	0.002

Overall one in ten children (10%) had witnessed their mother being abused by their father or another relative in the past month. Many of the children had also seen their father hit another man in the last month, 25.7% of boys and 17.9% of girls had done so. Boys and girls who had been victimized and who perpetrated violence themselves were significantly more likely to have witnessed abuse of their mother and seen their father fight (Table 2). They also had more conservative (patriarchal) gender attitudes.

The multinomial models of factors associated with peer violence are shown in Table 3, for each model the comparison category is no violence engagement. For girls, being more often hungry was associated with perpetration of peer violence, but not with victimization. Having the last day off school due to working, witnessing or hearing her father fight with another man and her having more patriarchal gender attitudes were all found to be significantly associated with both experiencing violence and perpetration.

**Table 3:** Multinomial regression models showing factors associated with girls' and boys’ engagement in and experience of peer violence.

	Victims v. non involved				Any perpetration v. not involved			
	RRR	95% CI		P value	RRR	95% CI		P value
**BOYS**								
**Two or more days absent from school in last 4 weeks**	3.96	1.85	8.47	<0.001	3.65	1.91	6.96	<0.001
**School performance**	0.79	0.64	0.98	0.032	0.67	0.55	0.82	<0.001
**Seen or heard father to have physical fight with another man**	3.42	0.76	15.36	0.108	8.85	2.10	37.28	0.003
**Gender inequitable attitudes**	1.13	1.01	1.26	0.032	1.19	1.07	1.33	0.001
**GIRLS**								
**Hunger score**	1.19	0.92	1.55	0.187	1.38	1.08	1.76	0.009
**Last day off due to work at home**	2.21	1.20	4.09	0.011	2.03	1.12	3.70	0.02
**Seen or heard father to have physical fight with another man**	2.14	1.01	4.53	0.047	5.73	2.89	11.34	<0.001
**Gender inequitable attitudes**	1.07	1.00	1.14	0.053	1.14	1.07	1.21	<0.001

For boys, missing two or more days of school and having poorer school performance were both associated with both violence victimization and perpetration. Hearing his father fight with another man was associated with peer violence perpetration. Having more patriarchal gender attitudes was also found to be significantly associated with both experiencing peer violence and perpetration.

The structural models for girls and boys perpetration of violence are presented in Figs 1 & 2 (and Table 4). For girls, there was one direct path between food insecurity (hunger) and peer violence perpetration, but four others were mediated by school performance (i.e. the less hungry girls had better school performance) and lower risk of perpetration, or by girl’s gender attitudes (i.e. hungrier girls had more patriarchal attitudes and perpetrated more, by abuse of their mother (i.e. hungrier girls had more abused mothers and more perpetration) and by their father fighting with other men (such that hungrier girls had fathers who fought more and were more likely to use violence themselves).

**Table 4:** Girls' and boys' perpetration path model: Direct effects, disturbance variances and equation-level goodness of fit.

**Parameter**	**Standardized coefficients**	**SE**	**z**	**P>|z|**	**[95% Conf. Interval]**		**Standardized coefficients**	**SE**	**z**	**P>|z|**	**[95% Conf. Interval]**	
**Direct effects**	**MODEL FOR BOYS**						**MODEL FOR GIRLS**					
Food insecurity—> gender attitudes	0.166	0.045	3.70	<0.0001	0.078	0.253	0.186	0.058	3.22	0.004	0.073	0.299
Food insecurity—> abuse of mother	0.194	0.047	4.16	<0.0001	0.103	0.285	0.257	0.062	4.15	<0.0001	0.135	0.379
Food insecurity—> father in fights	0.112	0.032	3.51	<0.0001	0.049	0.174	0.127	0.042	3.04	0.002	0.045	0.209
Food insecurity—> days off from school	0.241	0.059	4.06	<0.0001	0.124	0.358						
Food insecurity—> perpetration	0.130	0.053	2.45	0.014	0.026	0.234	0.116	0.046	2.52	0.012	0.026	0.207
Abuse of mother—> gender attitudes	0.145	0.047	3.09	0.002	0.053	0.238						
Gender attitudes—> perpetration	0.205	0.057	3.57	<0.0001	0.092	0.317	0.211	0.056	3.80	<0.0001	0.102	0.321
Abuse of mother—> perpetration	0.143	0.05	2.87	0.004	0.046	0.242	0.188	0.075	2.52	0.012	0.042	0.336
Father in fights—> perpetration	0.134	0.047	2.87	0.004	0.043	0.225	0.207	0.032	6.57	<0.0001	0.145	0.269
Days off from school—> perpetration	0.109	0.076	1.44	0.151	-0.040	0.258						
School performance—> perpetration	-0.137	0.061	-2.26	0.024	-0.265	-0.018	-0.166	0.054	-3.09	0.002	-0.271	-0.060
**Disturbance variances**	**Estimate**	**SE**			**[95% Conf. Interval]**		**Estimate**	**SE**			**[95% Conf. Interval]**	
Abuse of mother	0.962	0.018			0.927	0.998	0.934	0.032			0.873	0.999
Father in fights	0.988	0.007			0.974	1.002	0.984	0.011			0.963	1.005
Gender attitudes	0.942	0.018			0.901	0.985	0.965	0.022			0.924	1.008
School performance							0.991	0.009			0.974	1.008
Days absent from school	0.942	0.029			0.887	1.000						
Perpetration	0.816	0.045			0.731	0.911	0.777	0.061			0.666	0.905

For boys, there was one direct path between food insecurity (hunger) and peer violence perpetration, but four others were mediated by missing school for work or due to homework not being done (i.e. hungrier boys were more likely to miss school for these reasons and perpetrated more), or by boy’s gender attitudes (i.e. hungrier boys had more patriarchal attitudes and perpetrated more, by abuse of their mother (i.e. hungrier boys had more abused mothers and more perpetration) and by their father fighting with other men (such that hungrier boys had fathers who fought more and were more likely to use violence themselves). There was also a path between abuse of boys’ mother and his gender attitudes, such that those with more abused mothers had more patriarchal gender attitudes. Better school performance was linked to lower perpetration.

## Discussion

Peer violence is extremely common among girls and boys in grade 6 in Hyderabad schools and the prevalence reported appears to be considerably higher than that found in research from many other settings [6, 28]. These public school children come from poor urban slums which makes innocent and vulnerable exposed to the act of violence be it police brutality or feudal and landlords’ inhuman maltreatment to poor. Thus, we cannot ignore that violence is commercialized by the civil society nationally. Although some children had not been involved in the month prior to the interviews and some had only experienced violence as victims, about half of girls and three quarter of boys had themselves perpetrated and many had experienced violence as well as perpetration. The higher prevalence found among boys is in keeping with global patterns [18]. While, in the analysis we have sought to distinguish between the different groups of girls and boys by peer violence exposure category, there were very considerable similarities between the factors associated with peer violence in boys and girls, and most of the factors considered that showed an association in Tables 1 and 2 in fact had an elevated frequency for both victimization and perpetration (+/- victimization). This suggests predominant similarities between being a victim and perpetrator of peer violence and the interventions should focus on building resilience to all peer violence engagement.

Peer violence is often found to be associated with poverty [29], but the systematic review of other studies did not find that this was the case across studies, because in some settings those who perpetrate (and victims) are from wealthier families [14]. The ironical fact of poverty is that it not only gives hunger prangs but it also breeds aggression and depression in different forms and shapes. This is often fuelled by other socio economic factors as seen in our case study. Public schools in Pakistan unfortunately cater for economically deprived segment, which naturally are at the bottom of the social ladder. Thus, Hyderabad sample also inherits ills and woes of urbanization, a concrete jungle where other civic utilities are far from sufficient; water, electricity, sewerage, and lack of fresh air adds to the misery of urban slums and poor residential areas thus these factors act as suffocating for people living in urban slums. In Hyderabad schools the majority of girls and boys came from families experiencing some economic hardship, this study has shown a strong association between peer violence perpetration and food insecurity. In the multinomial model this was seen as significant for girls, after adjusting other factors. The Socio Economic Measurement (SEM) helps our understanding of how important food insecurity is in pathways to perpetration, as there are multiple paths between food insecurity and peer violence that are mediated by a range of different factors. The multinomial models show that among girls, being victimized and perpetrating, were twice as likely among those whose last day at school was missed due to work at home. Child labor is also rooted in poverty and has not, to our knowledge, previously been described in association with bullying, but this may reflect the individual research settings. Our findings are thus different from those of research in generally higher income settings [14].

Peer violence was associated with school attendance and performance. The research shows that school attendance was poor in the month before the study, with a very high proportion (over 80%) of all children missing two or more days. We have shown that a high proportion of students (about 1 in 5) missed school due to work in the month before the interviews, which highlights the high problem of child labor in the community. However days missed were higher among boys who experienced or were engaged in peer violence. The multinomial models show this to be higher for both victimized and perpetrating boys. This can be explained in the cultural milieu of Hyderabad where girls out of home visit is often limited to going schools but still they are exposed one way or the other to violence that is reflected in their acts. On the other hand, young boys are treated as helping hand with the parents at home and work, thus boys’ exposure and vulnerability is higher in Labour market also as compared to girls. Unfortunately, this is also reflected in boys’ behaviour both as perpetrators and victim.

Peer violence is particular associated with poorer school performance for girls and boys. This is seen for boys in the multinomial model, but for both girls and boys in the SEM. The path is direct for boys, but for girls it mediated a path between food insecurity and violence perpetration, such that food insecurity leads to poorer girls’ school performance. This findings has been shown in studies from a range of countries [30, 31]. One response from schools in this setting is corporal punishment. We show elsewhere connections between hunger, peer violence, poor academic performance and corporal punishment [32].

Previous research has pointed to the importance of exposure to family violence in perpetration of peer violence and points to both transmitted attitudes and values, as well as the psychological impact of witnessing violence [14, 33]. The impact of violence in the family on child engagement in violence is seen in associations between seeing or hearing the children’s father fighting with other men and violence directed at their mothers (both intimate partner and in-law perpetrated violence). Both of these are more common among children who are victimized than those not involved in violence, and more common among those who perpetrate. The multinomial regression model showed that having a father who had fought in the previous month was associated with more than a 5 (for girls) and 8 (for boys) times increased likelihood of the children perpetrating violence. The SEM showed that this behavior of fathers was strongly correlated to abuse of the mother and both of these variables mediated paths from food insecurity to violence perpetration.

We also looked at whether the gender attitudes held by children were associated with their involvement in peer violence. In keeping with the findings of research from Spain, our analysis shows that both boys and girls who were involved in violence held more patriarchal (gender inequitable) attitudes [34]. The multinomial models show these attitudes to be associated with both victim experience and perpetration for girls and boys. The SEM shows that for boys and girls gender attitudes mediated paths between food insecurity and violence. For further discussion of factors associated with gender attitudes are detailed elsewhere [35].

The model for boys further shows a path between abuse of the mother and gender attitudes, which suggests that for boys witnessing abuse of their mother is associated with developing more patriarchal attitudes. It seems likely that this is indicative of a constellation of ideas about gender and the use of violence which are learnt at home in households where the father (and his family) are violent and further, that in keeping with theorization about men’s violence and the links between ideas about gender, men’s use of violence against women and men’s use of violence with other men, we see these all interconnected [20, 36, 37]. Although in much of the research which has been conducted on gender violence by a male spouse against a female partner is regarded as ‘domestic violence’, in Asia it is often argued that violence by his relatives against the female spouse is very closely connected and should be equally regarded as part of the problem of ‘domestic violence’ [38]. Our findings would support this.

Our findings suggest that prevention of peer violence should include efforts to improve school performance and reduce absenteeism, these are major problems in schools in Pakistan and indeed the schools are often poorly resourced, teacher commitment to their work is highly variable and year overall is relatively short. Child labor seriously impacts school attendance, but in a community with highly prevalent food insecurity, prevention needs to be coupled to overall poverty reduction as well as changing parental attitudes towards the importance of education. In establishing the study we became aware that many schools in Hyderabad lacked basic amenities for children including drinking water and toilets, and school learning resources were very limited. Improving the quality of the school environment is crucial for overall promotion of learning.

The research has also shown the challenge of breaking with the social learning from the family around gender and the use of violence in reducing peer violence in school. Whilst this might involve a family-based intervention, research in other areas suggests that children can be empowered to rise above the problems and social norms from their home [21]. In fact on gender attitudes our children were already highly influenced by peers [35]. In this respect whole school interventions are more effective than classroom programmes or psychotherapeutic skills based [21]. This is crucial for building a future in Pakistan with more education, empowerment and less violence in the population. Our findings suggest that an intervention like that of the NGO Right To Play has some potential in achieving this, building on research which has shown that enhanced social and coping skills are protective [16, 39]. Evaluating its effectiveness is very important.

We were only able to assess engagement in peer violence in the previous month and some of the children who had not engaged in this period may have previously used violence. The same applied to violence used by fathers. Misclassification generally biased analyses towards the null. The analysis presented here has not focused on drivers of more severe violence and we have assumed that less severe and more severe are similar, but this may not be the case. Due to space limitations we have focused on drivers of any repeated violence. The study was not a population-based study and we thus are not able to present general population prevalence of violence in the lives of 11–13 year old in Hyderabad. Although volunteers different from the general population, our schools are generally representative of the public schools of the city and there is no reason to particularly expect biases in the schools participating in the trial or children in 6^th^ grade classes who volunteered which would invalidate the main analyses. Most of the schools and children who were invited to participate in the research agreed.

## Conclusion

This research shows the large percentage of youth engaged in peer violence, specifically almost half of girls and three quarters of boys reported perpetration against peers. Strong connections between peer violence and huger, poor academic performance, days of school missed, and witness of parental violence emerged. Food insecurity, notably 26% of the boys and 18% of the girls reported going to bed without dinner and similar percentages reported going to school without a meal. These same children were more likely to report perpetration and if a girl more perpetration and victimization were reported. While, in the analysis we have sought to distinguish between the different groups of girls and boys by peer violence exposure category, there were very considerable similarities between the factors associated with peer violence in boys and girls indicating interventions should be equally effective irrespective of gender. This research provides evidence foe a multi-prong approach to peer violence intervention that must include not only a curriculum for violence free relationships but also school feeding programs and community awareness of how witness to violence in the home is carried forth by youth to violence in the classroom.

To our knowledge this is the first study to describe prevalence, associated factors and drivers of peer violence from Pakistan, and the first from South or South East Asia. We have shown that peer violence among boys and girls in 6^th^ grade in Hyderabad, Pakistan schools is highly prevalent, indeed much more than in many settings. In contrast to research from wealthier countries, our analyses show poverty to be a key driver, and associated child labor. Further, we have shown that peer violence strongly influenced by the environment in the home with respect to the use of violence especially by fathers and other relatives against mothers and in fighting outside the home. This is associated with poor school performance and attendance, and patriarchal ideas about gender relations. The interconnectedness of violence use and experience points to the important challenge of preventing violence, especially in a society like Pakistan where there are enormous problems of violence in public life. An intervention with school children is not able to change all of the domains of a child’s life, but can empower children to be more assertive (in a positive sense) at school and at home, strengthen their performance at school and engagement with learning and through this change their engagement in peer violence. The intervention of Right to Play seeks to do this and its success will be evaluated through our trial.

## Supporting information

S1 File This file contains data collected from 1752 students. (SAV)

S2 File This file contains questionnaire in English language. (DOCX)

S3 File This file contains questionnaire used in local language Urdu. (PDF)

S4 File This file contains questionnaire used in local language Sindhi. (PDF)
