# Supplementary material for: Peer violence perpetration and victimization: Prevalence, associated factors and pathways among 1752 sixth grade boys and girls in schools in Pakistan
Source: PLoS One. 2017 Aug 17;12(8):e0180833. doi: 10.1371/journal.pone.0180833 (PMC5560651; doi:10.1371/journal.pone.0180833)
Supplement: S2 File — (DOCX) [file pone.0180833.s002.docx]

**PARTICIPANT INFORMATION SHEET**

| ID Number of Child |  |
| --- | --- |
| School Name |  |
| Class and Section |  |
| Name of Student |  |
| Name of Father |  |
| Name of Mother |  |
| Phone Number (Father) |  |
| Phone Number (Mother) |  |
| Residential Address |  |
| Phone Number (Self) |  |
| Phone Number  Name of Relative 1 |  |
| Phone Number  Name of Relative 2 |  |
| Phone Number  Name of Relative 3 |  |

| **PRACTICE QUESTIONS** | | | | | |
| --- | --- | --- | --- | --- | --- |
| PQ 1 | Do you live in Hyderabad? Yes = 1 No = 2 | | | | |
| PQ 2 | How many books do you have in your bag? _________ (Number) | | | | |
| Which statement best describes you: (CIRCLE ONE) | | | | | |
| PQ 3 | I like playing cricket everyday  I play cricket once in a while  I do not like to play cricket | | | | |
| PQ 4 | Do you like Shahid Afridi? Yes = 1 No = 2 | | | | |
| Please CIRCLE 0 if this Never Happened to you; CIRCLE 1 if it happened Once; CIRCLE 2 if it happened a Few Times; and CIRCLE 3 if it happened Many Times. | | | | | |
| How often in the past 4 weeks? | | **Never** | **Once** | **Few Times** | **Many Times** |
| PQ 5 | I have played Cricket | 0 | 1 | 2 | 3 |
| PQ 6 | Can you BOWL and BAT? | No = 0  Only bowl = 1  Only bat = 2  Bat and bowl = 3 | | | |

| Circle the number that describes how much you agree or disagree with each statement. | **Strongly Agree** | **Agree** | **Disagree** | **Strongly Disagree** |
| --- | --- | --- | --- | --- |

| PQ 7 | Stealing is OK | 1 | 2 | 3 | 4 |
| --- | --- | --- | --- | --- | --- |
| PQ 8 | Helping others is good | 1 | 2 | 3 | 4 |

| **DEMOGRAPHICS** | | |
| --- | --- | --- |
| D1 | Your Age in Years __________________________ | |
| D2 | Your Birthdate Month ______________ Date ___________ Year ___________ | |
| D3 | Are you a BOY =1 or GIRL=2 (CIRCLE ONE) | |
| D4 | Your Grade in School ___________________________________ | |
| D5 | How many people live in your home? | ____________(number) |
| D6 | How many brothers do you have? | ____________(number) |
| D7 | How many sisters do you have? | ____________(number) |

| **SCHOOL PERFORMANCE (SP)**  **Please CIRCLE one number that represents your grade in each school subject.** | | | | | |
| --- | --- | --- | --- | --- | --- |
| **How are you doing at school** | | | **FAIL** | **AVERAGE** | **Excellent** |
| SP1 | In reading and writing? | | 1 | 2 | 3 |
| SP2 | In Social studies? | | 1 | 2 | 3 |
| SP3 | In math? | | 1 | 2 | 3 |
| SP4 | In science? | | 1 | 2 | 3 |
| SP5a | Have you ever repeated a grade? CIRCLE ONE: Yes = 1 No=2 | | | | |
| SP 5b | If yes, what was the reason? CIRCLE ONE: Failed the class = 1 Changed school = 2 | | | | |
| SP6 | How many days of school did you miss last 4 weeks? Number __________________  (if you have gone to school on all days then move to CD 1) | | | | |
| SP7 | Thinking about the last time you missed a day from school, what was the PRIMARY reason? | SP7a You were ill ……………………….….. Yes = 1 No=2  SP7b Someone in the family was ill…………. Yes = 1 No=2  SP7c Lack of money for transport………..…. Yes = 1 No=2  SP7d Working at home ……….………….….. Yes = 1 No=2  SP7e Working to earn money………………… Yes = 1 No=2  SP7f Afraid to go to school due to bulling at school Yes = 1 No=2  SP7g Did not have homework done……………… Yes = 1 No=2  SP7h Did not want to go to school for another reason Yes = 1 No=2  SP7i If yes, please specify reason __________________ | | | |

| **Child Depression Self-Report**  **CIRCLE ONE sentence that describes you best for the past two weeks.** | |
| --- | --- |
| CD1  1  2  3 | I am sad once in a while.  I am sad many times.  I am sad all the time. |
| CD2  1  2  3 | Nothing will ever work out for me.  I am not sure if things will work out for me.  Things will work out for me OK. |
| CD3  1  2  3 | I do most things OK  I do many things wrong.  I do everything wrong. |
| CD4  1  2  3 | I have fun in many things.  I have fun in some things  Nothing is fun at all. |
| CD5  1  2  3 | I am important to my family  I am not sure if I am important to my family.  My family is better off without me. |
| CD6  1  2  3 | I hate myself  I do not like myself  I like myself |
| CD7  1  2  3 | All bad things are my fault  Many bad things are my fault  Bad things are not usually my fault |
| CD8  1  2  3 | I do not think about killing myself  I think about killing myself but would not do it.  I want to kill myself. |
| CD9  1  2  3 | I feel like crying every day  I feel like crying many days.  I feel like crying once in a while. |
| CD10  1  2  3 | I feel cranky all the time  I feel cranky many times  I am almost never cranky. |
| CD11  1  2  3 | I like being with people  I do not like being with people many times  I do not want to be with people at all. |
| **Child Depression Self-Report**  **CIRCLE ONE sentence that describes you best for the past two weeks.** | |
| CD12  1  2  3 | I cannot make up my mind about things  It is hard to make up my mind about things.  I make up my mind about things easily. |
| CD13  1  2  3 | I look ok  There are some bad things about my looks  I look ugly |
| CD14  1  2  3 | I have to push myself all the time to do my schoolwork  I have to push myself many times to do my schoolwork  Doing schoolwork is not a big problem |
| CD15  1  2  3 | I have trouble sleeping every night  I have trouble sleeping many nights  I sleep pretty well |
| CD16  1  2  3 | I am tired once in a while  I am tired many days  I am tired all the time |
| CD17  1  2  3 | Most days I do not feel like eating  Many days I do not feel like eating  I eat pretty well |
| CD18  1  2  3 | I do not worry about aches and pains  I worry about aches and pains many times  I worry about aches and pains all the time |
| CD19  1  2  3 | I do not feel alone  I feel alone many times  I feel alone all the time |
| CD20  1  2  3 | I never have fun at school  I have fun at school only once in a while  I have fun at school many times |
| CD21  1  2  3 | I have plenty of friends  I have some friends but I wish I had more  I do not have any friends |
| CD22  1  2  3 | My schoolwork is alright  My schoolwork is not as good as before  I do very badly in subjects I used to be good in |
| **Child Depression Self-Report**  **CIRCLE ONE sentence that describes you best for the past two weeks.** | |
| CD23  1  2  3 | I can never be as good as other kids  I can be as good as other kids if I want to  I am just as good as other kids |
| CD24  1  2  3 | Nobody really loves me  I am not sure if anybody loves me  I am sure that somebody loves me |
| CD25  1  2  3 | It is easy for me to get along with friends  I get into arguments with friends many times  I get into arguments with friends all the time |
| CD26  1  2  3 | I fall asleep during the day all the time  I fall asleep during the day many times  I almost never fall asleep during the day |
| CD27  1  2  3 | Most days I feel like I can’t stop eating  Many days I feel like I can’t stop eating  My eating is OK |
| CD28  1  2  3 | It is easy for me to remember things  It is a little hard to remember things  It is very hard to remember things |

| **Peer-Victimization Scale (PVS) (**Adapted from**:** Mynard, H. & Joseph, S. (2000). Development of the multidimensional peer-victimization scale. Aggressive Behavior, 26, 169-178) | | | | | |
| --- | --- | --- | --- | --- | --- |
| **Below is a list of things that some children do to other children. Please CIRCLE 0 if this Never Happened to you; CIRCLE if it happened Once; CIRCLE 2 if it happened a Few Times; and CIRCLE 3**  **if the behavior happened Many Times.** | | | | | |
| **How often within the past 4 weeks has another child done these things to you?** | | **Never** | **Once** | **Few Times**  **2or 3** | **Many Times**  **4 or more** |
| PVS1 | Called me bad names | 0 | 1 | 2 | 3 |
| PVS2 | Tried to get me into trouble with my friends | 0 | 1 | 2 | 3 |
| PVS3 | Took something of mine without permission | 0 | 1 | 2 | 3 |
| PVS4 | Made fun of me because of my appearance | 0 | 1 | 2 | 3 |
| PVS5 | Made fun of me for some reason apart from my appearance | 0 | 1 | 2 | 3 |
| PVS6 | Tripped me to make me fall | 0 | 1 | 2 | 3 |
| PVS7 | Pushed me to hurt me | 0 | 1 | 2 | 3 |
| PVS8 | Hurt me physically | 0 | 1 | 2 | 3 |
| PVS9 | Beat me so badly that I was injured | 0 | 1 | 2 | 3 |
| PVS10 | Deliberately broken something that belongs to me | 0 | 1 | 2 | 3 |
| PVS11 | Tried to make other children turn against me | 0 | 1 | 2 | 3 |
| PVS12 | Stole something from me | 0 | 1 | 2 | 3 |
| PVS13 | Refused to talk to me | 0 | 1 | 2 | 3 |
| PVS14 | Made other people not talk to me | 0 | 1 | 2 | 3 |
| PVS15 | Deliberately damaged something of mine | 0 | 1 | 2 | 3 |
| PVS16 | Swore at me | 0 | 1 | 2 | 4 |

| **Peer Victimization Locations (PVL)**  (Adapted from Swearer, S.M., & Cary, P.T. (2003). Perceptions and attitudes toward bullying in middle school youth: A developmental examination across the bully/victim continuum. Journal of Applied Psychology, 19, 63-79) | | | | | |
| --- | --- | --- | --- | --- | --- |
|  | **Where and how often did you experience these behaviors that other children did to you?** | **Never** | **Once** | **Few Times**  **2or 3** | **Many times**  **4 or more** |
| PVL1 | In a school classroom | 0 | 1 | 2 | 3 |
| PVL2 | In a school toilet area | 0 | 1 | 2 | 3 |
| PVL3 | In a school play ground | 0 | 1 | 2 | 3 |
| PVL4 | Outside school | 0 | 1 | 2 | 3 |
| PVL5 | In the streets | 0 | 1 | 2 | 3 |
| PVL6 | At home | 0 | 1 | 2 | 3 |
| PVL7 | How often the children/person who did these things to you was more powerful than you? | 0 | 1 | 2 | 3 |
| PVL8 | How often the children/person who did these things to you had many friends or was popular? | 0 | 1 | 2 | 3 |
| PVL9 | How often was the children/person who did these things to you older or larger than you? | 0 | 1 | 2 | 3 |
| PVL10 | Was it from boys or girls? **Circle One Answer**: Boys=1; Girls=2; Both Girls and Boys =3 | | | | |
| PVL11 | Was the person who did these things? **Circle One Answer**: Someone you know =1;  Someone you do not know =2; Both Someone you know and do not know = 3 | | | | |

| **Peer Victimization Impact (PVI)**  (Adapted from Swearer, S.M., & Cary, P.T. (2003). Perceptions and attitudes toward bullying in middle school youth: A developmental examination across the bully/victim continuum. Journal of Applied Psychology, 19, 63-79) | | | | | |
| --- | --- | --- | --- | --- | --- |
| How often did you experience the following problems because of these behaviors that other children did to you? | | **Never** | **Once** | **Few**  **Times**  **2 or 3** | **Many times**  4 or more |
| PVI1 | You felt sick | 0 | 1 | 2 | 3 |
| PVI2 | You could not make friends | 0 | 1 | 2 | 3 |
| PVI3 | You felt bad, sad, annoyed or helpless | 0 | 1 | 2 | 3 |
| PVI4 | You could not study or concentrate | 0 | 1 | 2 | 3 |
| PVI5 | You were absent from school | 0 | 1 | 2 | 3 |
| PVI6 | You had problems in relationships with your family | 0 | 1 | 2 | 3 |

| **CORPORAL PUNISHMENT SCHOOL (CPS)**  **How often within the past 4 weeks** | | **Never** | **Once** | **Few**  **Times**  2 or 3 | **Many times**  4 or more |
| --- | --- | --- | --- | --- | --- |
| CPS1 | Were you slapped, hit or beaten or otherwise physically punished by a teacher? | 0 | 1 | 2 | 3 |
| CPS2 | Did a teacher twist your ear? | 0 | 1 | 2 | 3 |
| CPS3 | Did a teacher make you stand on a bench? | 0 | 1 | 2 | 3 |
| CPS4 | Did a teacher make you run around as a punishment? | 0 | 1 | 2 | 3 |
| CPS5 | Did a teacher make you kneel down in class or outside? | 0 | 1 | 2 | 3 |
| CPS6 | In a past 4 weeks did a teacher hit you with a stick? | 0 | 1 | 2 | 4 |
| **PHYSICAL PUNISHMENT AT HOME (PPH)**  **How often within the past 4 weeks** | | **Never** | **Once** | **Few Times**  2 or 3 | **Many Times**  4 or more |
| PPH1 | Were you slapped, hit or beaten or otherwise physically punished by a parent? | 0 | 1 | 2 | 3 |
| PPH2 | In the past 4 weeks have you been beaten so hard at home that you were injured? | 0 | 1 | 2 | 3 |

| **Parent Fighting (PF) & Abuse of Mother**  **How often within the past 4 weeks** | | **Never** | **Once** | **Few**  **Times**  **2 or 3** | **Many Times**  4 or more |
| --- | --- | --- | --- | --- | --- |
| PF1 | Have you seen or heard that your father had a physical fight with another man? | 0 | 1 | 2 | 3 |
| PF2 | Have you seen or heard your father hit your mother? | 0 | 1 | 2 | 3 |
| PF3 | Have you seen or heard your mother being beaten by any of the family members? | 0 | 1 | 2 | 3 |
| PF4 | Does your father drink alcohol? Circle one: Yes=1; No=2 | | | | |

| **Peer-Perpetrator (PP) (**Adapted from**:** Mynard, H. & Joseph, S. (2000). Development of the multidimensional peer-victimization scale. Aggressive Behavior, 26, 169-178)  **Below is a list of things that some children do to other children. During the last 4 weeks, please CIRCLE 0 if you Never did this behavior to another child; CIRCLE 1 if you did the behavior Once; CIRCLE 2 if you did the behavior a Few Times; and CIRCLE 3 if it happened Many Times.** | | | | | |
| --- | --- | --- | --- | --- | --- |
| **How often within the past 4 weeks have you** | | **Never** | **Once** | **Few**  **Times**  2 or 3 | **Many Times**  4 or more |
| PP1 | Called another child bad names | 0 | 1 | 2 | 3 |
| PP2 | Tried to get another child into trouble with friends | 0 | 1 | 2 | 3 |
| PP3 | Upset or annoyed another child by taking something of theirs without permission | 0 | 1 | 2 | 3 |
| PP4 | Made fun of another child because of their appearance | 0 | 1 | 2 | 3 |
| PP5 | Made fun of another child for some reason apart from their appearance | 0 | 1 | 2 | 3 |
| PP6 | Tripped another child to make him or her fall | 0 | 1 | 2 | 3 |
| PP7 | Pushed another child to hurt him or her | 0 | 1 | 2 | 3 |
| PP8 | Hurt another child physically | 0 | 1 | 2 | 3 |
| PP9 | Beat another child so badly that they were injured | 0 | 1 | 2 | 3 |
| PP10 | Deliberately broken something that belong to another child | 0 | 1 | 2 | 3 |
| PP11 | Tried to make other children turn against another child | 0 | 1 | 2 | 3 |
| PP12 | Stolen something from another child | 0 | 1 | 2 | 3 |
| PP13 | Refused to talk to another child | 0 | 1 | 2 | 3 |
| PP14 | Made other children not talk to another child | 0 | 1 | 2 | 3 |
| PP15 | Deliberately damaged something of another child’s | 0 | 1 | 2 | 3 |
| PP16 | Swear at another child | 0 | 1 | 2 | 3 |

| **EARLY MARRIAGE (EM)** | |
| --- | --- |
| EM1 | Have you been promised in marriage to someone? Circle one: Yes=1 No=2 |
| EM2 | Has your family started other preparations for your marriage? Circle one: Yes=1 No=2 |
| EM3 | Thinking about the older sister or female cousin who is closest to your age, has she married? If Yes, What was her age at marriage Age___________________ |

| **GENDER ATTITUDES (GA)** (Developed by Afghanistan team) | | | | | |
| --- | --- | --- | --- | --- | --- |
| **Circle the number that describes how much you agree or disagree with each statement.** | | **Strongly Agree** | **Agree** | **Disagree** | **Strongly Disagree** |
| GA1 | I think girls in my family should go to school | 1 | 2 | 3 | 4 |
| GA2 | I think my father should give permission to my mother to go to the clinic | 1 | 2 | 3 | 4 |
| GA3 | I think my father should listen to my mother’s opinion on schooling | 1 | 2 | 3 | 4 |
| GA4 | I think my mother should have a say in how money is spent in my family | 1 | 2 | 3 | 4 |
| GA5 | I think my mother should be able to ask a religious scholar about solution of issues | 1 | 2 | 3 | 4 |
| GA6 | I think my father should respect the opinion of my mother on matters related to income generating work | 1 | 2 | 3 | 4 |
| GA7 | I think my father should be kind and caring toward my mother | 1 | 2 | 3 | 4 |
| GA8 | I think that my mother should always obey my father | 1 | 2 | 3 | 4 |
| GA9 | I think that my father has the right to punish my mother | 1 | 2 | 3 | 4 |

| **CHILD BEHAVIOR ATTITUDES (CBA)** (Developed by Afghanistan team) | | | | | |
| --- | --- | --- | --- | --- | --- |
| **Circle the number that describes how much you agree or disagree with each statement.** | | **Strongly Agree** | **Agree** | **Disagree** | **Strongly Disagree** |
| CBA1 | I think that if a child disobeys their parents they should be beaten | 1 | 2 | 3 | 4 |
| CBA2 | I think that if a child gets into fights their parents should beat them | 1 | 2 | 3 | 4 |
| CBA3 | I think that if a child talks back to their parents they should be punished by being beaten | 1 | 2 | 3 | 4 |
| **Circle the number that describes how much you agree or disagree with each statement.** | | **Strongly Agree** | **Agree** | **Disagree** | **Strongly Disagree** |
| CBA4 | I think a child who misbehaves at school should be beaten | 1 | 2 | 3 | 4 |
| CBA5 | I think that if a child hurts me I should hurts them back | 1 | 2 | 3 | 4 |

| **Women’s Participation (WP)** (Developed by Afghanistan team) | | | | | |
| --- | --- | --- | --- | --- | --- |
| **Circle the number that describes how much you agree or disagree with each statement.** | | **Strongly Agree** | **Agree** | **Disagree** | **Strongly Disagree** |
| WP1 | **I think women should be able to participate in**  Weddings | 1 | 2 | 3 | 4 |
| WP2 | Neighborhood events | 1 | 2 | 3 | 4 |
| WP3 | Skills training (e.g. computer skills, embroidery) | 1 | 2 | 3 | 4 |
| WP4 | Income generating activities | 1 | 2 | 3 | 4 |

| **Family Life (FL)** (Developed by Karachi team)  **Please circle ONE answer for each question:** | | | |
| --- | --- | --- | --- |
| FL1 | **In the last 4 weeks,** how often did you go to school without breakfast because of lack of food at home? | Never…………………..……...0  Sometimes…………..…………1  Every week……………………2  All or most days.……………..3 | |
| FL2 | **In the last 4 weeks,** how often do you go to sleep without dinner because of lack of food at home? | Never…………………..……...0  Sometimes…………..…………1  Every week…………………….2  All or most days.………………3 | |
| FL3 | Can your mother read **AND** write? | No………………………………0  She reads only………………….1  She reads and writes…………..2 | |
| FL4 | Can your father read **AND** write? | No………………………………0  He reads only………………….1  He reads and writes……………2 | |
| Please Circle YES OR NO to the questions below: | | YES = 1 | NO = 2 |
| FL5 | Do you have an electric fan at home? | YES = 1 | NO = 2 |
| FL6 | Do you have a refrigerator? | YES = 1 | NO = 2 |
| FL7 | Do you have sui gas for cooking? | YES = 1 | NO = 2 |
| FL8 | Do you get water at home? | YES = 1 | NO = 2 |
| FL9 | How many rooms do you have at home? | _________________(number) | |

| **DISABILITY QUESTIONS (DQ)** (The Washington Group Disability)  **Please circle ONE answer for each question:** | | |
| --- | --- | --- |
| DQ 1. | Do you have difficulty seeing, even if wearing glasses? | 1. No - no difficulty 2. Yes – some difficulty 3. Yes – a lot of difficulty 4. Cannot do at all |
| DQ 2 | Do you have difficulty hearing? | 1. No- no difficulty 2. Yes – some difficulty 3. Yes – a lot of difficulty 4. Cannot do at all |
| DQ 3. | Do you have difficulty walking or climbing steps? | 1. No- no difficulty 2. Yes – some difficulty 3. Yes – a lot of difficulty 4. Cannot do at all |
| DQ 4. | Do you have difficulty remembering or concentrating? | 1. No – no difficulty 2. Yes – some difficulty 3. Yes – a lot of difficulty 4. Cannot do at all |
| DQ 5 | Do you have difficulty speaking? | 1. No – no difficulty 2. Yes – some difficulty 3. Yes – a lot of difficulty 4. Cannot do at all |

.

THANK YOU FOR HELPING US TO HELP OTHER CHILDREN

PLEASE TURN THE PAGE FOR A GAME TO PLAY
