# Supplementary material for: Peer violence perpetration and victimization: Prevalence, associated factors and pathways among 1752 sixth grade boys and girls in schools in Pakistan
Source: PLoS One. 2017 Aug 17;12(8):e0180833. doi: 10.1371/journal.pone.0180833 (PMC5560651; doi:10.1371/journal.pone.0180833)
Supplement: S3 File — (PDF) [file pone.0180833.s003.pdf]

بچے کی معلومات

	شریک کار کا ID نمبر
	اسکول کا نام
	کلاس اور سیکشن
	بچے کا نام
	والد کا نام
	والدہ کا نام
	والد کا موبائل نمبر
	والدہ کا موبائل نمبر
	گھر کا پتہ
	خود کا موبائل نمبر
	پہلے قریبی رشتہ دار کا نمبر
	دوسرے قریبی رشتہ دار کا نمبر
	تیسرے قریبی رشتہ دار کا نمبر

Youth ID Number ____________________

Date ____________________

ابتدائی سوالات

PQ 1	کیا آپ حیدرآباد میں رہتے ہیں؟	1=ہاں 2= نہیں
PQ 2	آپ کے بستے میں کتنی کتابیں اور کاپیاں ہیں؟	____________________

کون سا جملہ آپ کی بہترین نمائندگی کرتا ہے؟(کسی ایک جملے پر دائرہ لگائیں)

	1	2	3
PQ 3	مجھے روز کرکٹ کھیلنا پسند ہے	مجھے کبھی کبھار کرکٹ کھیلنا پسند ہے	مجھے کرکٹ کھیلنا پسند بالکل نہیں ہے

PQ 4	کیا آپ کو شاہد خان آفریدی پسند ہیں؟	1=ہاں 2= نہیں

مندرجہ ذیل صورت اگر آپ کے ساتھ کبھی نہیں ہوئی ہے تو 0 پر دائرہ لگائیں، ایک دفعہ ہوئی ہے تو 1 پر دائرہ لگائیں، کئی دفعہ ہوئی ہے تو 2 پر دائرہ لگائیں اور بہت دفعہ ہوئی ہے تو پر 3 دائرہ لگائیں۔

	کتنی دفعہ پچھلے چار ہفتوں کے دوران:	کبھی نہیں	ایک دفعہ	کئی دفعہ	بہت دفعہ
PQ 5	آپ نے کرکٹ کھیلی۔	0	1	2	3

PQ 6	کیا آپ بولنگ اور بیٹنگ کرتے ہیں؟	نہیں = 0<br>صرف بولنگ = 1<br>صرف بیٹنگ = 2<br>بیٹنگ اور بولنگ۔ دونوں = 3

	مندرجہ ذیل جملوں سے آپ کس قدر اتفاق یا اختلاف کرتے ہیں۔ کسی ایک نمبر پر دائرہ لگائیں۔	سخت اتفاق	اتفاق	اختلاف	سخت اختلاف
PQ 7	چوری کرنا ٹھیک ہے۔	1	2	3	4
PQ 8	دوسروں کی مدد کرتا اچھی بات ہے۔	1	2	3	4

Youth ID Number ______________________

Date ______________________

DEMOGRAPHIC

D1 آپ کی عمر کیا ہے؟ ______________________

D2 آپ کی تاریخ پیدائش کیا ہے؟ (دن) __________ (مہینہ) __________ (سال) __________

D3 آپ کیا ہیں؟ (کسی ایک پر دائرہ لگائیے) لڑکا = 1 لڑکی = 2

D4 اسکول میں آپ کس جماعت میں پڑھتے ہیں؟ (کسی ایک پر دائرہ لگائیے)

چھٹی = 1 ساتویں = 2 آٹھویں = 3

D5 آپ کے گھر میں کتنے لوگ ہیں؟ ______________________

D6 آپ کے کتنے بھائی ہیں؟ ______________________

D7 آپ کی کتنی بہنیں ہیں؟ ______________________

Youth ID Number	______________________
Date	______________________

	مندرجہ ذیل مضامین میں آپ کی اسکول میں کارکردگی کیسی ہے؟	فیل	اوسط	عمدہ
SP 1	اردو میں یا سندھی میں	1	2	3
SP 2	سوشل سٹڈیز میں	1	2	3
SP 3	حساب میں	1	2	3
SP 4	سائنس میں	1	2	3
SP 5 (a)	کیا آپ نے کبھی کوئی کلاس دہرائی ہے؟	ہاں = 1		نہیں = 2
SP 5 (b)	اگر ہاں تو اس کی وجہ کیا تھی؟	فیل ہوئے = 1		اسکول تبدیل کی = 2
SP 6	پچھلے چار ہفتوں میں آپ کتنے دن اسکول نہیں آئے؟ ______________<br>(اگر آپ سب دن اسکول آئے ہیں تو CD1 پر چلے جائیں۔)			
SP 7 (a)	جن دنوں میں آپ اسکول نہیں آئے اس کی بنیادی وجہ کیا تھی؟<br>آپ بیمار تھے۔	ہاں = 1		نہیں = 2
SP 7 (b)	خاندان میں کوئی بیمار تھا / تھی۔	ہاں = 1		نہیں = 2
SP 7 (c)	اسکول آنے کے لئے سواری کے پیسے نہیں تھے۔	ہاں = 1		نہیں = 2
SP 7 (d)	گھر کے کام کر رہے تھے۔	ہاں = 1		نہیں = 2
SP 7 (e)	آمدنی / روزگار کمانے کے لئے گئے تھے۔	ہاں = 1		نہیں = 2
SP 7 (f)	اسکول جانے سے خوف زدہ تھے کیونکہ اسکول میں داداگیری ہوتی ہے۔	ہاں = 1		نہیں = 2
SP 7 (g)	ہوم ورک مکمل نہیں تھا۔	ہاں = 1		نہیں = 2
SP 7 (h)	کسی اور وجہ سے اسکول نہیں جانا چاہتے تھے۔	ہاں = 1		نہیں = 2
SP 7 (i)	اگر ہاں تو وجہ بتایئے۔ ______________			

Youth ID Number ____________________________

Date ____________________________

مندرجہ ذیل جملوں میں سے کسی ایک پر دائرہ لگائیں جو پچھلے دو ہفتوں کے دوران آپ کے بارے میں ٹھیک ہے۔		
CD1	1	میں کبھی کبھار اداس ہوتا / ہوتی ہوں۔
	2	میں اکثر اداس ہوتا / ہوتی ہوں۔
	3	میں ہمیشہ اداس ہوتا / ہوتی ہوں۔
CD2	1	میرے لئے کچھ بھی ٹھیک نہیں ہوتا۔
	2	مجھے یقین نہیں کہ سب کچھ میرے لئے ٹھیک رہے گا یا نہیں۔
	3	مجھے یقین ہے کہ سب کچھ میرے لئے ٹھیک رہے گا۔
CD3	1	میں بہت سارے کام ٹھیک کرتا / کرتی ہوں۔
	2	میں اکثر کام غلط کرتا / کرتی ہوں۔
	3	میں سارے کام غلط کرتا / کرتی ہوں۔
CD4	1	مجھے بہت سے کاموں میں مزا آتا ہے۔
	2	مجھے کچھ کاموں میں مزا آتا ہے۔
	3	مجھے کسی کام میں مزا نہیں آتا۔
CD5	1	میں اپنے خاندان کے لئے بہت اہم ہوں۔
	2	مجھے یقین نہیں کہ میں اپنے خاندان کے لئے اہم ہوں یا نہیں۔
	3	میرا خاندان میرے بغیر بہتر ہے۔
CD6	1	میں خود سے نفرت کرتا / کرتی ہوں۔
	2	میں خود کو پسند نہیں کرتا / کرتی ہوں۔
	3	میں خود کو پسند کرتا / کرتی ہوں۔
CD7	1	ساری بُری چیزیں میری غلطی ہیں۔
	2	بہت سی بُری چیزیں میری غلطی ہیں۔
	3	بُری چیزیں اکثر میری غلطی نہیں ہیں۔
CD8	1	میں خود کو جان سے مارنے کے بارے میں نہیں سوچتا / سوچتی۔
	2	میں خود کو جان سے مارنے کے بارے میں سوچتا / سوچتی ہوں مگر میں ایسا نہیں کروں گا / گی۔
	3	میں خود کو جان سے مارنا چاہتا / چاہتی ہوں۔
CD9	1	مجھے ہر روز رونے جیسا محسوس ہوتا ہے۔
	2	مجھے کئی روز رونے جیسا محسوس ہوتا ہے۔
	3	مجھے کبھی کبھار رونے جیسا محسوس ہوتا ہے۔
CD10	1	میں ہر وقت چڑ چڑاپن محسوس کرتا / کرتی ہوں۔
	2	میں اکثر چڑ چڑاپن محسوس کرتا / کرتی ہوں۔
	3	میں کبھی چڑ چڑاپن محسوس نہیں کرتا / کرتی ہوں۔

Youth ID Number ____________________

Date ____________________

		مندرجہ ذیل جملوں میں سے کسی ایک پر دائرہ لگائیں جو پچھلے دو ہفتوں کے دوران آپ کے بارے میں ٹھیک ہے۔
CD11	1	مجھے لوگوں کے ساتھ رہنا پسند ہے۔
	2	مجھے اکثر اوقات لوگوں کے ساتھ رہنا پسند نہیں ہے۔
	3	مجھے لوگوں کے ساتھ رہنا بالکل پسند نہیں ہے۔
CD12	1	میں فیصلے نہیں کر سکتا / سکتی۔
	2	میرے لئے فیصلے کرنا مشکل ہوتا ہے۔
	3	میں با آسانی فیصلے کرتا / کرتی ہوں۔
CD13	1	میں اچھا دکھتا / دکھتی ہوں۔
	2	میں تھوڑا بُرا دکھتا / دکھتی ہوں۔
	3	میں بدصورت دکھتا / دکھتی ہوں۔
CD14	1	مجھے اسکول کا کام کرنے کے لئے ہر وقت سخت محنت کرنی پڑتی ہے۔
	2	مجھے اسکول کا کام کرنے کے لئے اکثر سخت محنت کرنی پڑتی ہے۔
	3	میں اسکول کا کام با آسانی کرتا / کرتی ہوں۔
CD15	1	مجھے ہر رات بڑی مشکل سے نیند آتی ہے۔
	2	مجھے اکثر بڑی مشکل سے نیند آتی ہے۔
	3	مجھے پُر سکوں نیند آتی ہے۔
CD16	1	میں کبھی کبھار تھکن محسوس کرتا / کرتی ہوں۔
	2	میں اکثر تھکن محسوس کرتا / کرتی ہوں۔
	3	میں ہر وقت تھکن محسوس کرتا / کرتی ہوں۔
CD17	1	تقریباً ہر روز مجھے کھانا کھانے کا دل نہیں کرتا۔
	2	اکثر مجھے کھانا کھانے کا دل نہیں کرتا۔
	3	میری خوراک ٹھیک ہے۔
CD18	1	میں جسماتی درد کے لئے فکرمند نہیں ہوتا / ہوتی۔
	2	میں اکثر جسماتی درد کے لئے فکرمند ہوتا / ہوتی ہوں۔
	3	میں ہر وقت جسماتی درد کے لئے فکرمند ہوتا / ہوتی ہوں۔
CD19	1	میں اکیلاپن محسوس نہیں کرتا / کرتی۔
	2	میں اکثر اکیلاپن محسوس کرتا / کرتی ہوں۔
	3	میں ہر وقت اکیلاپن محسوس کرتا / کرتی ہوں۔
CD20	1	مجھے اسکول میں کبھی مزا نہیں آتا۔
	2	مجھے اسکول میں کبھی کبھار مزا آتا ہے۔
	3	مجھے اسکول میں ہر روز مزا آتا ہے۔

Youth ID Number	____________________
Date	____________________

مندرجہ ذیل جملوں میں سے کسی ایک پر دائرہ لگائیں جو پچھلے دو ہفتوں کے دوران آپ کے بارے میں ٹھیک ہے۔		
CD21	1	میرے بہت دوست ہیں۔
	2	میرے کئی دوست ہیں لیکن میری خواہش ہے کہ میرے کچھ اور دوست ہوں۔
	3	میرا کوئی دوست نہیں ہے۔
CD22	1	میرا اسکول کا کام اچھا ہے۔
	2	میرا اسکول کا کام پہلے جیسا اچھا نہیں ہے۔
	3	جن مضامین میں پہلے میں اچھا تھا اب میں اُن میں بہت بُرا ہوں۔
CD23	1	میں دوسرے بچوں جتنا اچھا نہیں بن سکتا۔
	2	اگر میں چاہوں تو میں دوسرے بچوں جتنا اچھا بن سکتا / سکتی ہوں۔
	3	میں بالکل دوسرے بچوں جتنا اچھا / جتنی اچھی ہوں۔
CD24	1	مجھے کوئی بھی حقیقت میں پیار نہیں کرتا۔
	2	مجھے یقین نہیں کہ کوئی مجھے پیار کرتا ہو۔
	3	مجھے یقین ہے کہ کوئی مجھے ضرور پیار کرتا ہو گا۔
CD25	1	میرے لئے آسان ہوتا ہے کہ میں دوستوں میں گھل مِل جاؤں۔
	2	میں اکثر دوستوں میں بحث میں لگ جاتا ہوں۔
	3	میں ہر وقت دوستوں میں بحث میں لگ جاتا ہوں۔
CD26	1	میں ہر وقت دن کے دوران سوتا / سوتی ہوں۔
	2	میں اکثر دن کے دوران سوتا / سوتی ہوں۔
	3	میں دن کے دورال کبھی نہیں سوتا / سوتی۔
CD27	1	تقریباً ہر روز میں محسوس کرتا / کرتی ہوں کہ میں کھانا کھانا نہیں روک سکتا / سکتی۔
	2	اکثر میں محسوس کرتا / کرتی ہوں کہ میں کھانا کھانا نہیں روک سکتا / سکتی۔
	3	میری خوراک ٹھیک ہے۔
CD28	1	میرے لئے چیزیں یاد رکھنا آسان ہے۔
	2	میرے لئے چیزیں یاد رکھنا مشکل ہے۔
	3	میرے لئے چیزیں یاد رکھنا انتہائی مشکل ہے۔

Youth ID Number ____________________

Date ____________________

بہت دفعہ	کچھ مرتبہ	ایک مرتبہ	کبھی نہیں	مندرجہ ذیل فہرست میں کچھ رویے اور عادات ہیں جو کچھ بچے دوسرے بچوں کے ساتھ کرتے ہیں۔ پچھلے چار ہفتوں کے دوران اگر آپ کے ساتھ کسی نے ایسا کبھی نہیں کیا تو 0 پر دائرہ لگائیں، اگر ایک مرتبہ آپ کے ساتھ ایسا ہوا ہے تو 1 پر دائرہ لگائیں، اگر کچھ مرتبہ آپ کے ساتھ ایسا ہوا ہے تو 2 پر دائرہ لگائیں اور اگر بہت دفعہ آپ کے ساتھ ایسا ہوا ہے تو 3 پر دائرہ لگائیں۔<br>پچھلے چار ہفتوں کے دوران دوسرے بچوں نے:	
3	2	1	0	مجھے بُرے ناموں سے پکارا / بلایا۔	PVS 1
3	2	1	0	مجھے میرے دوستوں کے ساتھ مشکل میں ڈالنے کی کوشش کی۔	PVS 2
3	2	1	0	میری کوئی چیز میری اجازت کے بغیر لے لی۔	PVS 3
3	2	1	0	میری ظاہری شکل و صورت کی وجہ سے میرا مزاق اڑایا۔	PVS 4
3	2	1	0	ظاہری شکل و صورت کے علاوہ کسی اور وجہ سے میرا مزاق اڑایا۔	PVS 5
3	2	1	0	مجھے گرانے کے لئے ٹنگڑی دی۔	PVS 6
3	2	1	0	مجھے چوٹ پہنچانے کے لئے مجھے دھکہ دیا۔	PVS 7
3	2	1	0	جسمانی طور پر مجھے چوٹ پہنچائی۔	PVS 8
3	2	1	0	مجھے اس قدر مارا کہ میں زخمی ہو گیا۔	PVS 9
3	2	1	0	میری کوئی چیز جان بوجھ کر توڑی۔	PVS 10
3	2	1	0	دوسرے بچوں کو میرے خلاف بھڑکایا۔	PVS 11
3	2	1	0	میری کوئی چیز چرائی۔	PVS 12
3	2	1	0	مجھ سے بات کرنے سے انکار کیا۔	PVS 13
3	2	1	0	دوسرے بچوں کو مجھ سے بات نہ کرنے پر مجبور کیا۔	PVS 14
3	2	1	0	جان بوجھ کر میری کوئی چیز تباہ کر دی۔	PVS 15
3	2	1	0	مجھے گالی دی۔	PVS 16

Youth ID Number ____________________________

Date ____________________________

بہت دفعہ	کچھ مرتبہ	ایک مرتبہ	کبھی نہیں	جن رویوں کا ہم نے ابھی ذکر کیا، آپ کے ساتھ کہاں اور کس قدر دوسرے بچوں نے یہ رویے اپنائے؟	
3	2	1	0	کلاس روم میں	PVL 1
3	2	1	0	اسکول کے بیت الخلا میں (toilet)	PVL 2
3	2	1	0	اسکول کے کھیل کے میدان میں	PVL 3
3	2	1	0	اسکول کے باہر	PVL 4
3	2	1	0	سڑک پر	PVL 5
3	2	1	0	گھر میں	PVL 6
3	2	1	0	جس انسان نے یہ رویہ آپ کے ساتھ اپنایا کتنی دفعہ وہ آپ سے زیادہ طاقتور تھا / تھی؟	PVL 7
3	2	1	0	کتنی دفعہ جس انسان نے یہ رویہ آپ کے ساتھ اپنایا اس کے زیادہ دوست تھے یا وہ بہت مشہور تھا / تھی؟	PVL 8
3	2	1	0	کتنی دفعہ جس انسان نے یہ رویہ آپ کے ساتھ اپنایا اس کی عمر آپ سے زیادہ تھی یا قد اور جسم میں آپ سے بڑا تھا / تھی؟	PVL 9

لڑکا	1	جس انسان نے یہ رویہ آپ کے ساتھ اپنایا وہ کون تھا / تھی؟	PVL 10
لڑکی	2		
دونوں	3		
آپ جانتے ہو۔	1	(کسی ایک نمبر پر دائرہ لگائیں) کیا اس شخص کو:	PVL 11
آپ نہیں جانتے۔	2		
آپ جانتے بھی ہو اور آپ نہیں بھی جانتے۔	3		

بہت دفعہ	کچھ مرتبہ	ایک مرتبہ	کبھی نہیں	مندرجہ ذیل تجربات / احساسات کتنی مرتبہ آپ کے ساتھ پیش آئے جب دوسرے بچوں نے آپ کے ساتھ یہ رویے رکھے؟	
3	2	1	0	آپ بیمار ہو گئے۔	PVI 1
3	2	1	0	آپ کو دوستی کرنے / دوست بنانے میں مشکل ہوئی۔	PVI 2
3	2	1	0	آپ کو بُرا، اداس، ناراض یا بے بس محسوس ہوا۔	PVI 3
3	2	1	0	آپ پڑھائی میں توجہ نہیں لگا سکے۔	PVI 4
3	2	1	0	آپ اسکول سے غیر حاضر رہے۔	PVI 5
3	2	1	0	آپ کے اپنے خاندان کے ساتھ آپ کے تعلقات متاثر ہوئے۔(مثلاً جو آپ کے ساتھ اسکول میں ہوا وہ آپ والدین کو نہیں بتا سکے)	PVI 6

**Youth ID Number**	____________________
**Date**	____________________

پچھلے چار ہفتوں کے دوران کتنی دفعہ آپ کو:		کبھی نہیں	ایک مرتبہ	کچھ مرتبہ	بہت دفعہ
CPS 1	استاد نے چماٹ ماری، مارا پیٹا یا جسمانی طور پر سزا دی۔	0	1	2	3
CPS 2	استاد نے آپ کا کان مروڑا۔	0	1	2	3
CPS 3	استاد نے آپ کو بینچ پر کھڑا کروایا۔	0	1	2	3
CPS 4	استاد نے آپ کو دوڑ لگانے کی سزا دی۔	0	1	2	3
CPS 5	استاد نے آپ کو کلاس کے اندر یا باہر مرغا بنوایا / گھٹنوں کے بل بٹھایا۔	0	1	2	3
CPS 6	استاد نے آپ کو چھڑی / اسٹک سے مارا۔	0	1	2	3

پچھلے چار ہفتوں کے دوران کتنی دفعہ:		کبھی نہیں	ایک مرتبہ	کچھ مرتبہ	بہت دفعہ
PPH 1	آپ کے والدین نے آپ کو چماٹ ماری، مارا پیٹا یا جسمانی طور پر سزا دی۔	0	1	2	3
PPH 2	آپ کو گھر پر اس قدر پیٹا گیا کہ آپ زخمی ہو گئے۔	0	1	2	3

پچھلے چار ہفتوں کے دوران کتنی دفعہ:		کبھی نہیں	ایک مرتبہ	کچھ مرتبہ	بہت دفعہ
PF 1	آپ نے دیکھا یا سنا ہے کہ آپ کے والد کی کسی دوسرے آدمی سے لڑائی ہوئی۔	0	1	2	3
PF 2	آپ نے دیکھا یا سنا ہے کہ آپ کے والد نے آپ کی والدہ کو مارا۔	0	1	2	3
PF 3	آپ نے دیکھا یا سنا ہے کہ آپ کے والدہ کو خاندان میں کسی اور نے مارا۔	0	1	2	3
PF 4	کیا آپ کے والد شراب پیتے ہیں؟	ہاں = 1		نہیں = 2	

Youth ID Number ____________________________

Date ____________________________

مندرجہ ذیل فہرست میں کچھ رویے اور عادات ہیں جو کچھ بچے دوسرے بچوں کے ساتھ کرتے ہیں۔ پچھلے چار ہفتوں کے دوران اگر آپ نے دوسرے بچوں کے ساتھ ایسا کبھی نہیں کیا تو 0 پر دائرہ لگائیں، اگر ایک مرتبہ آپ نے دوسرے بچوں کے ساتھ ایسا کیا ہے تو 1 پر دائرہ لگائیں، اگر کچھ مرتبہ آپ نے دوسرے بچوں کے ساتھ ایسا کیا ہے تو 2 پر دائرہ لگائیں اور اگر بہت دفعہ آپ نے دوسرے بچوں کے ساتھ ایسا کیا ہے تو 3 پر دائرہ لگائیں۔

پچھلے چار ہفتوں کے دوران آپ نے:

		کبھی نہیں	ایک مرتبہ	کچھ مرتبہ	بہت دفعہ
PP 1	دوسرے بچوں کو بُرے ناموں سے پکارا۔	0	1	2	3
PP 2	دوسرے بچوں کو دوستوں کے ساتھ مشکل میں لانے کی کوشش کی۔	0	1	2	3
PP 3	دوسرے بچوں کی کوئی چیز ان کی اجازت کے بغیر لے کر ان کو پریشان کیا۔	0	1	2	3
PP 4	دوسرے بچوں کا مزاق اڑایا ان کی ظاہری شکل و صورت کی وجہ سے۔	0	1	2	3
PP 5	ظاہری شکل و صورت کے علاوہ کسی اور وجہ سے دوسرے بچے کا مزاق اڑایا۔	0	1	2	3
PP 6	دوسرے بچوں کو گرانے کے لئے ٹنگڑی دی۔	0	1	2	3
PP 7	دوسرے بچوں کو چوٹ پہنچانے کے لئے انہیں دھکا دیا۔	0	1	2	3
PP 8	دوسرے بچوں کو جسمانی طور پر چوٹ پہنچائی۔	0	1	2	3
PP 9	دوسرے بچے کو اس طرح مارا کہ وہ زخمی ہو گیا۔	0	1	2	3
PP 10	جان بوجھ کر دوسرے بچوں کی کوئی چیز توڑی۔	0	1	2	3
PP 11	ایک بچے کو دوسرے بچے کے خلاف کیا۔	0	1	2	3
PP 12	دوسرے بچوں کی کوئی چیز چرائی۔	0	1	2	3
PP 13	دوسرے بچوں سے بات کرنے سے انکار کیا۔	0	1	2	3
PP 14	ایک بچے کو دوسرے بچے سے بات نہ کرنے پر مجبور کیا۔	0	1	2	3
PP 15	دوسرے بچوں کی کسی چیز کو جان بوجھ کر نقصان پہنچایا۔	0	1	2	3
PP 16	دوسرے بچوں کو گالی دی۔	0	1	2	3

Youth ID Number ______________________

Date ______________________

EM 1	کیا آپ کی کسی کے ساتھ شادی کرنے کا وعدہ کیا گیا ہے؟	ہاں = 1	نہیں = 2
EM 2	کیا آپ کے خاندان میں آپ کی شادی کی دیگر تیاریاں شروع کر دی گئی ہیں؟	ہاں = 1	نہیں = 2
EM 3	اگر آپ کے خاندان میں آپ کی بڑی بہن یا لڑکی رشتہ دار جن کی عمر تقریباً آپ جتنی ہے، کیا ان کی شادی ہو چکی ہے؟<br>اگر ہاں تو شادی کے وقت ان کی عمر کیا تھی؟ ______________	ہاں = 1	نہیں = 2

	اس نمبر پر دائرہ لگائیں جس قدر آپ مندرجہ ذیل جملوں سے اتفاق یا اختلاف کرتے ہیں:	سخت اتفاق	اتفاق	اختلاف	سخت اختلاف
GA 1	میرے خیال سے میرے خاندان کی لڑکیوں کو اسکول جانا چاہیے۔	1	2	3	4
GA 2	میرے خیال سے میرے والد کو میری والدہ کو کلینک جانے کی اجازت دینی چاہیے۔	1	2	3	4
GA 3	میرے خیال سے میرے والد کو بچوں کی اسکول کی تعلیم کے بارے میں میری والدہ کی رائے سننی چاہیے۔	1	2	3	4
GA 4	میرے خیال سے میری والدہ کو یہ حق ہو کہ وہ اس باری میں بات کریں کہ خاندان میں پیسے کس طرح خرچ کرنے ہیں۔	1	2	3	4
GA 5	میرے خیال سے میری والدہ مذہبی عالم سے مسائل کا حل پوچھ سکیں۔	1	2	3	4
GA 6	میرے خیال سے میرے والد کو آمدنی کے ذرائع اور کام سے متعلق معاملات میں میری والدہ کی رائے سننی چاہیے۔	1	2	3	4
GA 7	میرے خیال سے میرے والد کو میری والدہ کے ساتھ رحم دل اور خیال رکھنے والا ہونا چاہیے۔	1	2	3	4
GA 8	میرے خیال سے میری والدہ کو میرے والد کی ہمیشہ فرماں بردار ہونا چاہیے۔	1	2	3	4
GA 9	میرے خیال سے میرے والد کو میری والدہ کو سزا دینے کا حق ہے۔	1	2	3	4

Youth ID Number ____________________________

Date ____________________________

سخت اختلاف	اختلاف	اتفاق	سخت اتفاق	اس نمبر پر دائرہ لگائیں جس قدر آپ مندرجہ ذیل جملوں سے اتفاق یا اختلاف کرتے ہیں:	
4	3	2	1	میرے خیال سے اگر بچہ / بچی والدین کی نافرمانی کرتا / کرتی ہے تو والدین کو اسے مارنا چاہیے۔	CBA 1
4	3	2	1	میرے خیال سے اگر بچہ / بچی والدین سے لڑائی کرتا / کرتی ہے تو والدین کو اسے مارنا چاہیے۔	CBA 2
4	3	2	1	میرے خیال سے اگر بچہ / بچی والدین کو سامنے جواب دیتا / دیتی ہے تو والدین کو اسے مارنا چاہیے۔	CBA 3
4	3	2	1	میرے خیال سے اگر بچہ / بچی اسکول میں بد سلوکی کرے تو اسے مارنا چاہیے۔	CBA 4
4	3	2	1	میرے خیال سے اگر ایک بچہ / بچی مجھے نقصان پہنچاتا / پہنچاتی ہے تو مجھے بھی اسے نقصان پہنچانا چاہیے۔	CBA 5

سخت اختلاف	اختلاف	اتفاق	سخت اتفاق	اس نمبر پر دائرہ لگائیں جس قدر آپ مندرجہ ذیل جملوں سے اتفاق یا اختلاف کرتے ہیں:<br>میں سمجھتا / سمجھتی ہوں کہ ایک عورت کو مندرجہ ذیل چیزوں میں شرکت کرنی چاہیے:	
4	3	2	1	شادیوں میں۔	WP 1
4	3	2	1	پڑوس کی تقاریب میں۔	WP 2
4	3	2	1	ہنر حاصل کرنے کی تربیتوں میں۔(کمپیوٹر، سلائی، کڑھائی)	WP 3
4	3	2	1	آمدنی بڑھانے کی سرگرمیوں میں۔	WP 4

**Youth ID Number**	________________________
**Date**	________________________

مندرجہ ذیل سوالات میں کسی ایک پر دائرہ لگائیں:			
FL 1	پچھلے چار ہفتوں میں کتنی دفعہ آپ اسکول ناشتہ کئے بغیر گئے کیوں کہ گھر پر کھانے کی کمی تھی؟	کبھی نہیں= 0<br>کبھی کبھار= 1<br>ہفتے میں ایک دفعہ= 2<br>ہمیشہ= 3	
FL 2	پچھلے چار ہفتوں میں کتنی دفعہ آپ رات کا کھانا کھائے بغیر سو گئے کیوں کہ گھر پر کھانے کی کمی تھی؟	کبھی نہیں= 0<br>کبھی کبھار= 1<br>ہفتے میں ایک دفعہ= 2<br>ہمیشہ= 3	
FL 3	کیا آپ کی امی لکھ اور پڑھ سکتی ہیں؟	نہیں= 0<br>صرف پڑھ سکتی ہیں= 1<br>لکھ اور پڑھ سکتی ہیں= 2	
FL 4	کیا آپ کے ابّو لکھ اور پڑھ سکتے ہیں؟	نہیں= 0<br>صرف پڑھ سکتے ہیں= 1<br>لکھ اور پڑھ سکتے ہیں= 2	
FL 5	کیا آپ کے گھر میں بجلی سے چلنے والا پنکھا ہے؟	ہاں = 1	نہیں = 2
FL 6	کیا آپ کے گھر میں فرج (fridge) ہے؟	ہاں = 1	نہیں = 2
FL 7	کیا آپ کے گھر میں کھانا پکانے کے لئے سوئی گیس ہے؟	ہاں = 1	نہیں = 2
FL 8	کیا آپ کے گھر میں پانی آتا ہے؟	ہاں = 1	نہیں = 2
FL 9	آپ کے گھر میں کتنے کمرے ہیں؟ ____________________		

	مندرجہ ذیل سوالات میں کسی ایک پر دائرہ لگائیں:	نہیں۔ کوئی مشکل پیش نہیں آتی	ہاں۔ کچھ مشکل پیش آتی ہے	ہاں۔ بہت زیادہ مشکل پیش آتی ہے	کچھ بھی نہیں کر سکتا
DQ 1	کیا آپ کو عینک پہننے کے باوجود بھی دیکھنے میں مشکل پیش آتی ہے؟	1	2	3	4
DQ 2	کیا آپ کو سننے میں مشکل پیش آتی ہے؟	1	2	3	4
DQ 3	کیا آپ کو پیدل چلنے یا سیڑھیاں چڑھنے میں مشکل پیش آتی ہے؟	1	2	3	4
DQ 4	کیا آپ کو زبانی یاد کرنے یا توجہ دینے میں مشکل پیش آتی ہے؟	1	2	3	4
DQ 5	کیا آپ کو بات کرنے میں مشکل پیش آتی ہے؟	1	2	3	4

شکریہ
