# Supplementary material for: Peer violence perpetration and victimization: Prevalence, associated factors and pathways among 1752 sixth grade boys and girls in schools in Pakistan
Source: PLoS One. 2017 Aug 17;12(8):e0180833. doi: 10.1371/journal.pone.0180833 (PMC5560651; doi:10.1371/journal.pone.0180833)
Supplement: S4 File — (PDF) [file pone.0180833.s004.pdf]

Youth ID Number ___________________________

Date ___________________________

ٻار جي معلومات

ٻار جو ID نمبر	
اسڪول جو نالو	
ڪلاس ۽ سيڪشن	
ٻار جو نالو	
پيءُ جو نالو	
ماءُ جو نالو	
پيءُ جو فون نمبر	
ماءُ جو فون نمبر	
گهر جو پتو	
پنهنجو فون نمبر	
مائٽ جو فون نمبر۱	
مائٽ جو فون نمبر۲	
مائٽ جو فون نمبر۳	

Youth ID Number ____________________________

Date ____________________________

ابتدائي سوالُ

PQ 1	چا اوهين حيدرآباد ۾ رهندا آهيو؟ — ها = 1 — نہ = 2
PQ 2	اوهان جي بستي ۾ ڪيترين ڪتابون ۽ ڪاپيون آهن؟ ____________________________
	هيٺ ڏنل جملن مان ڪنهن هڪڙي نمبر تي دائرو لڳايو جيڪو توهان جي باري ۾ گزريل ہہ هفتن لاءِ ٺيڪ هجي
PQ 3	
1	مون کي روز ڪرڪٽ کيڏڻ پسند آهي.
2	مون کي ڪڏهن ڪڏهن ڪرڪٽ کيڏڻ پسند آهي.
3	مون کي ڪرڪٽ کيڏڻ پسند ڪون آهي.
PQ 4	چا اوهين شاهد آفريدي کي پسند ڪندا آهيو؟ — ها = 1 — نہ = 2

		ڪڏهن بہ نہ	هڪ دفعو	ڪجھ دفعا	گھڻا دفعا
	گزريل چار هفتن جي دوران اوهان ڪڏهن بہ اهڙو نہ ڪيو آهي تہ 0 تي دائرو لڳايو، اگر اوهان هڪ ڀيرو ڪيو آهي تہ 1 تي، اگر ڪجھ مرتبہ ڪيو آهي تہ 2 تي ۽ اگر گھڻي دفعہ ڪيو آهي تہ 3 تي دائرو لڳايو.				
PQ 5	مان ڪرڪٽ کيڏي.	0	1	2	3

PQ 6	اوهين بيٽنگ ۽ بالنگ ڪندا / ڪندي آهيو؟	نہ = 0<br>رڳو بالنگ = 1<br>رڳو بيٽنگ = 2<br>بيٽنگ ۽ بالنگ = 3

		سخت اتفاق	اتفاق	اختلاف	سخت اختلاف
	انهي جملي کي پڙهي هن نمبر تي دائرو لڳايو جنهن سان اوهان اتفاق يا اختلاف رکو ٿا				
PQ 7	چوري ڪرڻ جائز آهي.	1	2	3	4
8 PQ	پين جي مدد ڪرڻ سٺي عادت آهي.	1	2	3	4

**Youth ID Number**	____________________________
**Date**	____________________________

DEMOGRAPHIC

D1 اوهان جي عمر ڇا آهي؟ ________________________________

D2 اوهان جي تاريخ پيدائش ڇا آهي؟ ڏينهن _________ مهينو ___________ سال ___________

D3 اوهان ڪير آهيو؟

(ڪنهن هڪ تي دائرو لڳايو) 1: ڇوڪرو 2: ڇوڪري

D4 اسڪول ۾ اوهين ڪهڙي ڪلاس ۾ پڙهندا آهيو؟

(ڪنهن هڪ تي دائرو لڳايو) 1: ڇهين 2: ستين 3: ائين

D5 اوهان جي گهر ۾ ڪيترا ڀاتي آهن ________________________________

D6 اوهان جا ڪيترا ڀائر آهن؟ ________________________________

D7 اوهان جون ڪيتريون ڀيڻون آهن؟ ________________________________

Youth ID Number ____________________

Date ____________________

	هيٺ ڏنل مضمونن ۾ اوهان اسڪول ۾ ڪيئن آهيو؟	فيل	وچولو	سٺو
SP 1	اردو يا سنڌي ۾	1	2	3
SP 2	سوشل اسٽڊيز ۾	1	2	3
SP 3	رياضي ۾	1	2	3
SP 4	سائنس ۾	1	2	3
SP 5 (a)	ڪڏهن توهان ڪلاس دهرايو آهي؟	ها = 1		نہ = 2
SP 5 (b)	اگر ها تہ ڇو؟	1 = فيل ٿيا هئا		2 = اسڪول تبديل ڪيو هو
SP 6	گزريل ڇئن هفتن ۾ اوهين ڪيترا ڏينهن اسڪول ڪونہ ويا؟ ____________________<br>(اگر توهين سڀ ڏينهن اسڪول ۾ موجود هئا تہ CD 1 تي وڃو)			
SP 7 (a)	اوهين بيمار هئا	ها = 1		نہ = 2
SP 7 (b)	خاندان ۾ ڪو بيمار هو / هئي	ها = 1		نہ = 2
SP 7 (c)	اسڪول اچڻ لاء سواري لاء پيسہ نہ هئا	ها = 1		نہ = 2
SP 7 (d)	گهر جا ڪم ڪندا هئا	ها = 1		نہ = 2
SP 7 (e)	آمدني / روزگار لاء ويندا هئا	ها = 1		نہ = 2
SP 7 (f)	اسڪول وڃڻ لاء خوف زده هئا جو اتي دادا گيري ٿيندي آهي	ها = 1		نہ = 2
SP 7 (g)	اوهان جو هوم ورڪ مڪمل نہ هو	ها = 1		نہ = 2
SP 7 (h)	ڪنهن ٻئي سبب جي ڪري اسڪول نہ ويا	ها = 1		نہ = 2
SP 7 (i)	اگرها تہ سبب ٻڌايو ____________________			

Youth ID Number ____________________________

Date ____________________________

هيٺ ڏنل جملن مان ڪنهن هڪڙي تي دائرو لڳايو جيڪو توهان جي باري ۾ گزريل ٻن هفتن جي باري ۾ ٺيڪ هجي	
مان ڪڏهن ڪڏهن اداس ٿيندو آهيان<br>مان گهڻو ڪري اداس رهندو آهيان<br>مان سدائين اداس رهندو آهيان	CD1<br>1<br>2<br>3
منهنجي لاءِ ڪڏهن بہ ڪجھ ٺيڪ نٿو ٿئي<br>مون کي يقين ڪونهي تہ منهنجي لاءِ ڪجھ ٺيڪ ٿيندو<br>مون کي يقين آهي تہ منهنجي لاءِ سڀ ٺيڪ ٿي ويندو	CD2<br>1<br>2<br>3
مان گهڻا ڪم سٺا ڪريان ٿو / ٿي<br>مان اڪثر ڪم غلط ڪريان ٿو / ٿي<br>مان سڀ ڪم غلط ڪريان ٿو / ٿي	CD3<br>1<br>2<br>3
مون کي گهڻو ڪري ڪمن ۾ مزو ايندو آهي<br>مون کي ڪجھ ڪمن ۾ مزو ايندو آهي<br>مون کي ڪنهن بہ ڪم ۾ مزو نہ ايندو آهي	CD4<br>1<br>2<br>3
مان منهنجي ڪٽنب / خاندان لاءِ اهم آهيان<br>مون کي يقين ڪونهي تہ مان منهنجي ڪٽنب / خاندان لاءِ اهم آهيان يا نہ<br>منهنجو ڪٽنب / خاندان مون کان بغير ڀلو آهي	CD5<br>1<br>2<br>3
مان پنهنجي پاڻ کان نفرت ڪريان ٿو / ٿي<br>مان پنهنجي پاڻ کي پسند نٿو / نٿي ڪريان<br>مان پنهنجي پاڻ کي پسند ڪريان ٿو / ٿي	CD6<br>1<br>2<br>3
تمام خراب شيون منهنجي ڪري ٿين ٿيون<br>گهڻو ڪري خراب شيون منهنجي ڪري ٿين ٿيون<br>خراب شيون اڪثر منهنجي ڪري نٿيون ٿين	CD7<br>1<br>2<br>3
مان پنهنجي زندگي کي ختم ڪرڻ بابت نٿو / نٿي سوچان<br>مان پنهنجي زندگي کي ختم ڪرڻ بابت سوچان ٿو پر مان ائين نہ ڪندس<br>مان پنهنجي زندگي کي ختم ڪرڻ چاهيان ٿو / ٿي	CD8<br>1<br>2<br>3
مان هر وقت روئان ٿو / ٿي<br>مان اڪثر روئان ٿو / ٿي<br>مان ڪڏهن ڪڏهن روئان ٿو / ٿي	CD9<br>1<br>2<br>3
مان هر وقت چڙچڙو رهندو / رهندي آهيان<br>مان اڪثر چڙچڙو رهندو / رهندي آهيان<br>مان ڪڏهن بہ چڙچڙو ڪونہ رهندو / رهندي آهيان	CD10<br>1<br>2<br>3

Youth ID Number __________________________

Date __________________________

هيٺ ڏنل جملن مان ڪنهن هڪڙي تي دائرو لڳايو جيڪو توهان جي باري ۾ گزريل ٻن هفتن جي باري ۾ ٺيڪ هجي	
مون کي ماڻهن سان گڏ رهڻ پسند آهي<br>مون کي اڪثر ماڻهن سان گڏ رهڻ پسند ڪونهي<br>مون کي ماڻهن سان گڏ رهڻ بالڪل پسند ڪونهي	CD11<br>1<br>2<br>3
مان فيصلا نٿو / نٿي ڪري سگهان<br>منهنجي لاءِ فيصلا ڪرڻ مشڪل آهن<br>مان آساني سان فيصلا ڪري سگهان ٿو / ٿي	CD12<br>1<br>2<br>3
مان سهڻو / سهڻي لڳان ٿو / ٿي<br>مان ڏسڻ ۾ ٿورڙو بدصورت لڳان ٿو / ٿي<br>مان ڏسڻ ۾ بالڪل بدصورت لڳان ٿو / ٿي	CD13<br>1<br>2<br>3
مون کي اسڪول جو ڪم ڪرڻ لاءِ هر وقت گهڻي محنت ڪرڻي پوي ٿي<br>مون کي اسڪول جو ڪم ڪرڻ لاءِ اڪثر گهڻي محنت ڪرڻي پوي ٿي<br>مان اسڪول جو ڪم آساني سان ڪندو / ڪندي آهيان	CD14<br>1<br>2<br>3
مون کي هر رات سمهڻ ۾ ڏکائي ٿئي ٿي<br>مون کي اڪثر سمهڻ ۾ ڏکيائي ٿيئ ٿي<br>مان سڪون سان سمهان ٿو / ٿي	CD15<br>1<br>2<br>3
مان ڪڏهن ڪڏهن ٿڪل محسوس ڪريان ٿو / ٿي<br>مان اڪثر ٿڪل محسوس ڪريان ٿو / ٿي<br>مان هر وقت ٿڪل محسوس ڪريان ٿو / ٿي	CD16<br>1<br>2<br>3
گهڻو ڪري هر روز مون کي کاڌو کائڻ نٿو وڻي<br>اڪثر مون کي کاڌو کائڻ نٿو وڻي<br>منهنجي خوراڪ ٺيڪ آهي	CD17<br>1<br>2<br>3
مان جسماني سورن لاءِ پريشان نٿو/نٿي ٿيان<br>مان اڪثر جسماني سورن لاءِ پريشان ٿيان ٿو / ٿي<br>مان هر وقت جسماني سورن لاءِ پريشان ٿيان ٿو / ٿي	CD18<br>1<br>2<br>3
مان اڪيلائي محسوس نٿو / نٿي ڪريان<br>مان اڪثر اڪيلائي محسوس ڪريان ٿو / ٿي<br>مان هر وقت اڪيلائي محسوس ڪريان ٿو / ٿي	CD19<br>1<br>2<br>3
مون کي ڪڏهن بہ اسڪول ۾ مزو نٿو اچي<br>مون کي ڪڏهن ڪڏهن اسڪول ۾ مزو اچي ٿو<br>مون کي گهڻو ڪري اسڪول ۾ مزو اچي ٿو	CD20<br>1<br>2<br>3

Youth ID Number ___________________________

Date ___________________________

هيٺ ڏنل جملن مان ڪنهن هڪڙي تي دائرو لڳايو جيڪو توهان جي باري ۾ گزريل ٻن هفتن جي باري ۾ ٺيڪ هجي	
	CD21
منهنجا گهڻا دوست آهن	1
منهنجا ڪجهه دوست آهن پر منهنجي خواهش آهي ته گهڻا دوست هجن	2
منهنجو ڪو به دوست ڪون آهي	3
	CD22
منهنجو اسڪول جو ڪم سٺو آهي	1
منهنجو اسڪول جو ڪم پهرين وانگر سٺو ڪونهي	2
جنهن سبق ۾ مان پهرين سٺو هئس هاڻي مان ان ۾ خراب آهيان	3
	CD23
مان ڪڏهن به ٻين ٻارن جهڙو سٺو نٿو / نٿي ٿي سگهان	1
مان ڪوشش ڪريان ته ٻين ٻارن جهڙو سٺو ٿي سگهان ٿو / ٿي	2
مان ٻين ٻارن جهڙو سٺو / جهڙي سٺي آهيان	3
	CD24
مون کي ڪير به پيار نٿو ڪري	1
مون کي يقين ڪونهي ته ڪير مون کي پيار ڪري ٿو يا نه	2
مون کي يقين آهي ته ڪو نه ڪو مون کي ضرور پيار ڪندو هوندو	3
	CD25
مان آساني سان دوستن ۾ ملي ويندو / ويندي آهيان	1
مان اڪثر دوستن سان بحث ۾ پئجي ويندو / ويندي آهيان	2
مان هميشه دوستن سان بحث ۾ پئجي ويندو / ويندي آهيان	3
	CD26
مان ڏينهن جي دوران هر وقت سمهان ٿو / ٿي	1
مان ڏينهن جي دوران اڪثر سمهان ٿو / ٿي	2
مان ڏينهن جي دوران ڪڏهن به نٿو / نٿي سمهان	3
	CD27
مان گهڻو ڪري پاڻ کي کائڻ کان روڪي نٿو / نٿي سگهان	1
مان اڪثر پاڻ کي کائڻ کان روڪي نٿو / نٿي سگهان	2
منهنجي خوراڪ ٺيڪ آهي	3
	CD28
منهنجي لاءِ شيون ياد ڪرڻ آسان آهن	1
منهنجي لاءِ شيون ياد ڪرڻ ٿورڙو مشڪل آهن	2
منهنجي لاءِ شيون ياد ڪرڻ گهڻو مشڪل آهن	3

Youth ID Number ______________________

Date ______________________

گھڻا دفعا	ڪجھ دفعا	هڪ دفعو	ڪڏهن به نه	هيٺ ڏنل فهرست ۾ ڪجھ رويا ۽ عادتون آهن جيڪي ڪجھ ٻار ٻين ٻارن سان ڪندا آهن. گزريل ڇئن هفتن جي دوران اوهان سان ڪنهن به اهڙو نہ ڪيو آهي تہ 0 تي دائرو لڳايو، اگر اوهان سان هڪ ڀيرو ٿيو آهي تہ 1 تي، اگر ڪجھ مرتبہ ٿيو آهي تہ 2 تي ۽ اگر گھڻا دفعا ٿيو آهي تہ 3 تي دائرو لڳايو.<br>گزريل ڇئن هفتن جي دوران ٻين ٻارن:	
3	2	1	0	مون کي خراب نالن سان سڏيو هو	PVS 1
3	2	1	0	مون کي پنهنجي دوستن سان مشڪل ۾ وجهڻ جي ڪوشش ڪئي وئي	PVS 2
3	2	1	0	منهنجي ڪا شيءِ منهنجي اجازت کان بغير کئي ويئي	PVS 3
3	2	1	0	منهنجي شڪل و صورت جو مزاق اڏايو	PVS 4
3	2	1	0	شڪل و صورت جي علاوه ڪنهن ٻئي سبب ڪري مزاق اڏايو ويو	PVS 5
3	2	1	0	مون کي ٽنگڙي ڏيئي ڪيرايو ويو	PVS 6
3	2	1	0	مون کي ڌِڪو ڏيئي ڪيرايو ويو	PVS 7
3	2	1	0	جسماني طور تي چوٽ پهچائي وئي	PVS 8
3	2	1	0	مون کي ايترو ماريو ويو جو آءُ زخمي ٿي ويس	PVS 9
3	2	1	0	مون جي ڪا شئي ڄاڻي ٻجهي ٽوڙي وئي	PVS 10
3	2	1	0	ٻين ٻارن کي منهنجي خلاف ڀڙڪايو ويو	PVS 11
3	2	1	0	منهنجي ڪا شئي چوري ڪئي وئي	PVS 12
3	2	1	0	مون سان ڳالهائڻ کان انڪار ڪيو ويو	PVS 13
3	2	1	0	ٻين ٻارن کي مون سان نہ ڳالهائڻ لاءِ مجبور ڪيو ويو	PVS 14
3	2	1	0	ڄاڻي ٻجهي منهنجي ڪا شئي تباه ڪئي وئي	PVS 15
3	2	1	0	مون کي گار ڏني وئي	PVS 16

Youth ID Number ____________________________

Date ____________________________

	اوهان سان ڪٿي ۽ ڪيتري دفعا هي رويو رکيو:	ڪڏهن به نه	هڪ دفعو	ڪجھ دفعا	گھڻا دفعا
PVL 1	ڪلاس روم ۾	0	1	2	3
PVL 2	اسڪول جي بيت الخلاء ۾ (TOILET)	0	1	2	3
PVL 3	اسڪول جي راند جي ميدان ۾	0	1	2	3
PVL 4	اسڪول جي ٻاهر	0	1	2	3
PVL 5	رستي تي	0	1	2	3
PVL 6	گھر ۾	0	1	2	3
PVL 7	ڪيتري دفعا وڌيڪ جنهن اوهان سان هيئ رويو رکيو هو اهو اوهان سان طاقتور هو؟	0	1	2	3
PVL 8	ان جا الاهي دوست هوا يا هو مشهور آهي؟	0	1	2	3
PVL 9	ڪيتري دفعا جنهن اوهان سان هيئ رويو رکيو هو ان ماڻهو جي عمر توهان کان وڌيڪ هئي يا هو قد ۽ جسم ۾ اوهان کان وڏو هو؟	0	1	2	3
PVL 10	هو ڇوڪرن يا ڇوڪرين مان هو؟	ڇوڪرو 1<br>ڇوڪري 2<br>ٻئي 3			
PVL 11	ان ماڻهو ڪي اوهين	سڃاڻو ٿا 1<br>سڃاڻو نٿا 2<br>ٻئي 3			

	هيٺ ڏنل تجربات/احساسات ڪيترا دفعا اوهان سان پيش آيا؟	ڪڏهن به نه	هڪ دفعو	ڪجھ دفعا	گھڻا دفعا
PVI 1	اوهين بيمار تي ويا	0	1	2	3
PVI 2	اوهان کي دوست ٺائڻ ۾ مشڪل ٿئي	0	1	2	3
PVI 3	اوهان کي خراب، اداس، ناراض محسوس ٿيو	0	1	2	3
PVI 4	اوهين پڙهائي ۾ ڌيان نہ ڏئي سگھيا	0	1	2	3
PVI 5	اوهين اسڪول مان غيرحاضر رهيا	0	1	2	3
PVI 6	اوهان جا تعلقات ڪتنب سان مشڪل ٿيا (اوهان سان اسڪول ۾ جيڪو ٿيو اهو پهنجي ماءُ ۽ پيءُ کي ٻڌائي نہ سگھيا)	0	1	2	3

Youth ID Number ______________________________

Date ______________________________

گھڻا دفعا	ڪجھ دفعا	هڪ دفعو	ڪڏهن بہ نہ	گزريل چئن هفتن ۾ ڪيترا دفعا؟	
3	2	1	0	استاد چماٽ هنئي، ماريو يا جسماني سزا ڏني	CPS 1
3	2	1	0	استاد اوهان جو ڪن مروڙيو	CPS 2
3	2	1	0	استاد اوهان کي بينچ تي بيهاريو	CPS 3
3	2	1	0	استاد اوهان کي ڊوڙڻ جي سزا ڏني	CPS 4
3	2	1	0	استاد اوهان کي ڪلاس جي اندر يا ٻاهر مرغو بڻايو/ گوڏن تي ويهاريو	CPS 5
3	2	1	0	استاد اوهان کي ڏنڊي سان ماريو	CPS 6

گھڻا دفعا	ڪجھ دفعا	هڪ دفعو	ڪڏهن بہ نہ	گزريل چئن هفتن ۾ ڪيترا دفعا؟	
3	2	1	0	اوهان جي والدين اوهان کي چماٽ هنئي، ماريو، يا جسماني طور سزا ڏني	PPH 1
3	2	1	0	اوهان کي گھر ۾ ايترو ماريو جو اوهين زخمي ٿي ويا	PPH 2

گھڻا دفعا	ڪجھ دفعا	هڪ دفعو	ڪڏهن بہ نہ	گزريل چئن هفتن ۾ ڪيترا دفعا؟	
3	2	1	0	اوهان ڏٺو يا ٻڌو تہ اوهان جي والد جي ڪنهن ٻئي ماڻهو سان لڙائي ٿي	PF 1
3	2	1	0	اوهان ڏٺو يا ٻڌو تہ ڪيترا دفعا اوهان جي والد اوهان جي والده کي ماريو	PF 2
3	2	1	0	اوهان ڏٺو يا ٻڌو تہ اوهان جي والده کي خاندان مان ڪنهن ماريو	PF 3
نہ = 2		ها = 1		چا اوهان جو والد شراب پئي ٿو؟	PF 4

Youth ID Number ______________________________

Date ______________________________

هيٺ ڏنل فهرست ۾ ڪجھ رويا ۽ عادتون آهن جو ڪجھ ٻار ٻين ٻارن سان ڪندا آهن. گزريل چار هفتن جي دوران اوهان سان ڪنهن بہ اهڙو نہ ڪيو آهي تہ 0 تي دائرو لڳائيو، اگر اوهان سان هڪ ڀيرو ٿيو آهي تہ 1 تي، اگر ڪجھ مرتبہ ٿيو آهي تہ 2 تي ۽ اگر گهڻي دفعہ ٿيو آهي تہ 3 تي دائرو لڳائيو.<br>گزريل چئن هفتن جي دوران اوهان:		ڪڏهن بہ نہ	هڪ دفعو	ڪجھ دفعا	گهڻا دفعا
PP 1	ٻين ٻارن کي بري نالي سان سڏيو	0	1	2	3
PP 2	ٻين ٻارن کي دوستن سان گڏ مشڪل ۾ وجهڻ جي ڪوشش ڪئي	0	1	2	3
PP 3	ٻين ٻارن کي پريشان ۽ تنگ ڪيو (انهن جي ڪا شيءِ بغير) اجازت ڪڍي ورتي	0	1	2	3
PP 4	ٻين ٻارن جو مزاق اڏايو انهن جي شڪل وصورت جي ڪري	0	1	2	3
PP 5	ظاهري شڪل و صورت جي علاوه ڪنهن ٻيئي سبب ٻين ٻارن جو مزاق اڏايو	0	1	2	3
PP 6	ٻين ٻارن کي ڪيرائڻ لاءِ ٽنگڙي ڏني	0	1	2	3
PP 7	ٻين ٻارن کي چوٽ پهچائڻ لاءِ ڌڪو ڏنو	0	1	2	3
PP 8	ٻين ٻارن کي جسماني طور تي چوٽ پهچائي	0	1	2	3
PP 9	ٻين ٻارن کي ماري زخمي ڪيو	0	1	2	3
PP 10	ڄاڻي ٻجهي ٻين ٻارن جون شيون ٽوڙي ڇڏيون	0	1	2	3
PP 11	هڪ ٻار کي ٻيئي ٻار جي خلاف ڪيو	0	1	2	3
PP 12	ٻين ٻارن جي ڪا شيءِ چوري ڪئي	0	1	2	3
PP 13	ٻين ٻارن سان ڳالهائڻ کان انڪار ڪيو	0	1	2	3
PP 14	هڪ ٻار کي ٻيئي ٻار سان نہ ڳالهائڻ لاءِ مجبور ڪيو / اڪسايو	0	1	2	3
PP 15	ٻيئي ٻار جي ڪنهن شيءِ کي ڄاڻي ٻجهي نقصان پهچايو	0	1	2	3
PP 16	ٻين ٻارن کي گاريون ڏنيون	0	1	2	3

Youth ID Number ____________________________

Date ____________________________

نہ = 2	ها = 1	چا اوهان جي شادي ڪنهن سان ڪرائڻ جو وعدو ڪيو ويو آهي؟	EM 1
نہ = 2	ها = 1	چا اوهان جي شادي جي تياري شروع ٿي وئي آهي؟	EM 2
نہ = 2	ها = 1	چا اوهان جي خاندان ۾ اوهان جي وڏي ڀيڻ يا ڪا ڇوڪري رشتہ دار جنهن جي عمر اوهان جيتري آهي ڇا هن جي شادي ٿي وئۓ آهي؟<br><br>اگرها تہ شادي وقت ان جي عمر ڇا هئي؟ ____________	EM 3

سخت اختلاف	اختلاف	اتفاق	سخت اتفاق	انهي جملي کي پڙهي ان نمبر تي دائرو لڳايوجنهن سان اوهان اتفاق يا اختلاف رکو ٿا	
4	3	2	1	منهنجي خيال ۾ اسان جي خاندان ۾ ڇوڪرين کي اسڪول وڃڻ گهرجي	GA 1
4	3	2	1	منهنجي خيال ۾ اسان جي خاندان ۾ مڙس کي زال کي ڪلينڪ وڃڻ جي اجازت ڏيڻ گهرجي	GA 2
4	3	2	1	منهنجي خيال ۾ منهنجي خاندان جي مڙسن کي ٻارن کي اسڪول جي تعليم جي بابت انهن جي زالن جي راءِ ٻڌڻ گهرجي	GA 3
4	3	2	1	منهنجي خيال ۾ اسان جي خاندان ۾ زالن کي حق هئڻ گهرجي تہ خاندان ۾ پيسو ڪيئن خرچ ڪرڻ گهرجي	GA 4
4	3	2	1	منهنجي خيال ۾ اسان جي خاندان ۾ زالون مذهبي عالم کان مسئلن جو حل پڇي ٿي سگهن	GA 5
4	3	2	1	منهنجي خيال ۾ اسان جي خاندان ۾ مڙسن کي آمدني جي زريئن ۽ ڪم سان متعلق معاملات ۾ زالن جي راءِ ٻڌڻ گهرجي	GA 6
4	3	2	1	منهنجي خيال ۾ منهنجي خاندان جي مڙسن کي پنهنجي زالن سان رحمدل ۽ خيال رکندڙ هئڻ گهرجي	GA 7
4	3	2	1	منهنجي خيال ۾ زالن کي پنهنجي مڙسن جو هميشہ فرمانبردار هئڻ گهرجي	GA 8
4	3	2	1	منهنجي خيال ۾ جڏهن ڪا زال ڪجهہ غلط ڪري تہ مڙس کي ان کي سزا ڏيڻ جوحق هجي	GA 9

Youth ID Number ____________________________

Date ____________________________

	هيٺين جملن کي پڙهي ان نمبر تي دائرو لڳايو جنهن سان اوهان اتفاق يا اختلاف رکو ٿا	سخت اتفاق	اتفاق	اختلاف	سخت اختلاف
CBA 1	منهنجي خيال ۾ اگر ٻار والدين جي نافرماني ڪن ٿا ته والدين کي انهن کي مارڻ گهرجي	1	2	3	4
CBA 2	منهنجي خيال ۾ اگر ٻار والدين سان لڙائي ڪن ٿا ته والدين کي انهن کي مارڻ گهرجي	1	2	3	4
CBA 3	منهنجي خيال ۾ اگر ٻار والدين کي سامهون جواب ڏين ٿا ته والدين کي انهن کي مارڻ گهرجي	1	2	3	4
CBA 4	منهنجي خيال ۾ اگر ٻار اسڪول ۾ بدتميزي/بد سلوڪي ڪن ٿا ته انهن کي مارڻ گهرجي	1	2	3	4
CBA 5	منهنجي خيال ۾ اگر ڪو ٻار مون کي نقصان پهنچائي ٿو ته مون کي به ان کي نقصان پهچائڻ گهرجي	1	2	3	4

	هيٺين جملن کي پڙهي هن نمبر تي دائرو لڳايو جنهن سان اوهان اتفاق يا اختلاف رکو ٿا آءُ سمجهان ٿو ته زالن کي شرڪت ڪرڻ گهرجي:	سخت اتفاق	اتفاق	اختلاف	سخت اختلاف
WP 1	شادين ۾	1	2	3	4
WP 2	پاڙي وارن جي تقريبن ۾	1	2	3	4
WP 3	هنر/ مهارت حاصل ڪرڻ جي تربيتن ۾ (ڪمپيوٽر ۽ سلائي ڪڙهائی)	1	2	3	4
WP 4	آمدني وڌائڻ / ڪمائڻ جي سرگرمين ۾	1	2	3	4

Youth ID Number ______________________

Date ______________________

هيٺ ڏنل سوالن مان ڪنهن هڪ جواب تي دائرو لڳايو			
FL 1	گزريل چئن هفتن ۾ ڪيتري دفعه اوهين اسڪول ناشتي جي بغيرويا چاڪاڻ جو گهر ۾ کاڌي جي ڪمي هئي؟	ڪڏهن نه =0<br>ڪڏهن ڪڏهن =1<br>هر هفتي =2<br>هميشه =3	
FL 2	گزريل چئن هفتن ۾ ڪيترا دفعا اوهين رات جو کاڌو کائڻ بغير سمهي رهيا چاڪاڻ جو گهر ۾ کاڌي جي ڪمي هئي؟	ڪڏهن نه =0<br>ڪڏهن ڪڏهن =1<br>هر هفتي =2<br>هميشه =3	
FL 3	چا اوهان جي ماءُ لکي پڙهي سگهي ٿي؟	نه =0<br>رڳو پڙهي سگهي ٿي =1<br>لکي پڙهي سگهي ٿي =2	
FL 4	چا اوهان جو پيُ لکي پڙهي سگهي ٿو؟	نه =0<br>رڳو پڙهي سگهي ٿو =1<br>لکي پڙهي سگهي ٿو =2	
FL 5	چا اوهان جي گهر ۾ بجلي سان هلڻ وارو پکو آهي؟	ها = 1	نه = 2
FL 6	چا اوهان جي گهر ۾ فرج آهي؟	ها = 1	نه = 2
FL 7	چا اوهان جي گهر ۾ کاڌو رڌڻ لاءِ سوئي گيس آهي؟	ها = 1	نه = 2
FL 8	چا اوهان جي گهر ۾ پاڻي اچي ٿو؟	ها = 1	نه = 2
FL 9	اوهان جي گهر ۾ ڪيترا ڪمرا آهن؟ ______________		

	هيٺ ڏنل سوالن مان ڪنهن هڪ جواب تي دائرو لڳايو	نه . ڪا به مشڪل نٿي ٿيئي	ها . ٿوري مشڪل ٿيئي ٿي	ها . گهڻي مشڪل ٿيئي ٿي	ڪجهه به نٿو / نٿي ڪري سگهان
DQ 1	چا اوهان کي عينڪ پائڻ کان پوءِ به ڏسڻ ۾ مشڪل ٿيئي ٿي؟	1	2	3	4
DQ 2	چا اوهان کي ٻڌڻ ۾ مشڪل ٿيئي ٿي؟	1	2	3	4
DQ 3	چا اوهان کي پيدل هلڻ يا چاڙهيون چڙهندي مشڪل ٿيئي ٿي؟	1	2	3	4
DQ 4	چا اوهان کي زباني ياد ڪرڻ يا توجه ڏيڻ ۾ مشڪل ٿيئي ٿي؟	1	2	3	4
DQ 5	چا اوهان کي ڳالهائڻ ۾ مشڪل ٿيئي ٿي؟	1	2	3	4

شڪريه
